# Task-specific effects of noisy galvanic vestibular stimulation on gait symmetry in adolescent idiopathic scoliosis

**DOI:** 10.7717/peerj.21947

**Published:** 2026-11-12

**Authors:** Yan Li, Zhou Zhang, Ailipinai Yasen, Xumiao Peng, Yuzi Miao, Surong Qian, Haoyu Xie

**Affiliations:** 1 Rehabilitation Medicine Center, The Second Affiliated Hospital of Wenzhou Medical University, Wenzhou, Zhejiang, China; 2 Department of Rehabilitation Medicine, First Affiliated Hospital of Sun Yat-Sen University, Guangzhou, Guangdong, China; 3 Department of Rehabilitation Medicine, The Affiliated Suzhou Hospital of Nanjing Medical University, Suzhou Municipal Hospital, Gusu School, Nanjing Medical University, Suzhou, Jiangsu, China; 4 Rehabilitation Medicine Center, First Affiliated Hospital of Nanjing Medical University, Nanjing, Jiangsu, China

**Keywords:** Noisy galvanic vestibular stimulation, Adolescent idiopathic scoliosis, Stair negotiation, Gait symmetry

## Abstract

**Background:**

Vestibular dysfunction has been identified as a potential contributor to postural instability and gait asymmetry in adolescent idiopathic scoliosis (AIS). Noisy galvanic vestibular stimulation (nGVS) enhances gait performance by optimizing vestibular signal processing *via* stochastic resonance. However, how nGVS modulates gait symmetry in AIS and whether its efficacy depends on locomotor tasks or spinal deformity severity remain to be elucidated. This study aimed to investigate the effects of nGVS on medial-lateral gait symmetry in patients with AIS during level walking and single-step stair negotiation.

**Methods:**

This single-group crossover study recruited 30 patients with AIS to perform level walking, stair ascent, and stair descent under nGVS, sham-stimulation, and no-stimulation conditions. Primary dependent measures included medial-lateral gait symmetry across kinematic (lateral trunk displacement (T), lateral trunk lean (α), lateral pelvic displacement (P), pelvic obliquity (β), and hip adduction (θ)) and kinetic domains (first (ML1) and second (ML2) medial-lateral ground reaction force peaks), quantified by the symmetry index (SI) and normalized cross-correlation coefficient (Xcorr). *Post-hoc* comparisons prioritized the (nGVS *vs.* sham) contrast to isolate true nGVS efficacy. Correlations between the sham-controlled net efficacy (Δ) and Cobb angle were also examined.

**Results:**

Compared to sham stimulation, nGVS significantly increased Xcorr_α (descent: *p* = 0.017; ascent: *p* = 0.033), Xcorr_β (descent: *p* = 0.006), Xcorr_θ (ascent: *p* = 0.008), and Xcorr_ML (descent: *p* < 0.001), while decreasing SI_ML1 (descent: *p* = 0.015; ascent: *p* = 0.022) during single-step stair negotiation. Correlation analyses revealed that greater Cobb angle was significantly correlated with Δ Xcorr_ML (descent: *ρ* = 0.583, *p* = 0.001; ascent: *r* = 0.575, *p* = 0.001), Δ Xcorr_α (descent: *r* = 0.539, *p* = 0.002; ascent: *ρ* = 0.427, *p* = 0.019), Δ Xcorr_β (descent: *ρ* = 0.394, *p* = 0.031), Δ Xcorr_θ (ascent: *r* = 0.398, *p* = 0.030), and Δ SI_ML1 (descent: *ρ* =  − 0.444, *p* = 0.014). However, this state-dependent relationship did not reach statistical significance across Δ SI_α (*r* =  − 0.394, *p* = 0.137) and Δ SI_β (*r* =  − 0.423, *p* = 0.108).

**Conclusions:**

These findings demonstrated that nGVS ameliorated medial-lateral gait symmetry in AIS by refining multi-segmental control under heightened biomechanical demands, indicating a task-specific pattern. Furthermore, nGVS-induced enhancements in medial-lateral gait symmetry were state-dependent, wherein patients exhibiting larger Cobb angles within the mild-to-moderate range (10°–30°) derived greater biomechanical gains.

## Introduction

Adolescent idiopathic scoliosis (AIS), the most prevalent musculoskeletal disorder during puberty, is a complex three-dimensional spinal deformity of unknown origin (Wong & Chiu, 2025). It is primarily characterized by a spinal curvature in the frontal plane, which compromises medial-lateral postural stability (Koura et al., 2025). This instability triggers compensatory postural adaptations (*e.g.*, trunk displacement and pelvic obliquity), which subsequently result in a consistent postural asymmetry (Farshad, Catanzaro & Schmid, 2016). Since normal gait requires integrated postural control, these aberrant postural adaptations in AIS inevitably lead to gait deficits and asymmetry, with a high prevalence of up to 85%, posing increased risk of falling and significant challenges to activities of daily living in AIS (Paradkar et al., 2025; Şahin, Urak & Akkaya, 2019). Multiple studies have reported greater asymmetry in patients with AIS during level walking compared to healthy individuals (Ji et al., 2024; Wu et al., 2019; Yang et al., 2013). Such side-to-side gait asymmetries can be comprehensively evaluated at two distinct levels: single discrete values (typically kinematic or kinetic peaks) and continuous movement trajectories (Wu et al., 2019). The symmetry index (SI) is one of the most widely used metrics for quantifying bilateral gait asymmetries in patients with AIS (Ji et al., 2024). Its primary advantages lie in its computational simplicity and intuitive interpretability, where 0% indicates perfect symmetry and values approaching 100% reflect greater asymmetry (Xie & Chien, 2024). However, SI is inherently limited by its sole reliance on discrete peak values, thereby failing to capture parameter dynamics throughout the gait cycle (Daryabor et al., 2017). To overcome this limitation, the cross-correlation coefficient (CC) is employed to capture continuous parameter variations and assess overall trajectory alignment (Yang et al., 2013). Furthermore, to address the correlation inaccuracies from waveform magnitude, normalized CC (Xcorr) is calculated to evaluate waveform similarity across the gait cycle (Ogihara et al., 2020). Therefore, combining both SI and Xcorr allows for a holistic assessment of gait symmetry (Wang, Chien & He, 2025).

Normal gait symmetry relies on the multisensory integration of visual, proprioceptive, and vestibular feedback, with the vestibular apparatus serving as a critical internal reference frame for spatial orientation and dynamic gait control (Peterka, 2002). Accumulating neurophysiological evidence demonstrates structural and functional abnormalities of the vestibular system in AIS cohorts, which potentially contribute to their sensorimotor deficits (Hawasli, Hullar & Dorward, 2015). Multiple publications have reported lateral semicircular canal asymmetry (Carry et al., 2020; Hitier et al., 2015) and increased distance between superior and lateral semicircular canals (Cortés-Pérez et al., 2022) in scoliotic adolescents compared to healthy controls, constituting the morphological foundation of vestibular dysfunction. Moreover, several studies have employed posturography in patients with AIS and observed impaired postural control during eyes-closed standing tasks, where reliance on vestibular feedback is heightened, as evidenced by greater medial-lateral displacement and net force (Hatzilazaridis et al., 2019; Pialasse et al., 2015a; Pialasse et al., 2015b; Pialasse et al., 2016). Notably, spinal deformity severity, quantified by the Cobb angle, has been shown to be associated with the magnitude of vestibular dysfunction. Specifically, Vlădăreanu et al. (2024) recruited 89 scoliotic adolescents with Cobb angles ranging from 10° to 30°, and found that those with vestibular dysfunction (positive history of motion sickness and positive Fukuda stepping test) exhibited significantly greater Cobb angles. Collectively, vestibular dysfunction represents a key pathophysiological mechanism underlying AIS and is associated with spinal deformity severity.

Vestibular dysfunction in AIS further leads to impaired multisensory integration and sensory reweighting within the central nervous system (CNS), thereby compromising motor outputs. Pialasse et al. (2015b) utilized galvanic vestibular stimulation to elicit vestibular-evoked postural responses and observed altered sensory reweighting in patients with AIS, as evidenced by a significantly larger weight allocated to vestibular feedback (13.17%), compared to healthy adolescents (6.03%). Beyond static postural control, such vestibular dysfunction and aberrant multisensory integration also underlie the gait deficits in AIS. Mallau et al. (2007) reported that scoliotic adolescents exhibited significantly impaired spatiotemporal gait characteristics and compromised yaw/roll-plane head-trunk stabilization, particularly during high-demanding tasks (*e.g.*, walking on a beam). Additionally, Park et al. (2016) established a direct association between spinal deformity severity and gait asymmetry, demonstrating a positive correlation between Cobb angles and SI of ground reaction force (GRF) peak magnitudes during level walking. These findings raise a critical question: can enhancing vestibular feedback in patients with AIS improve gait symmetry?

Noisy galvanic vestibular stimulation (nGVS) has emerged as a safe, non-invasive neuromodulation technique to modulate vestibular signals (Herssens & McCrum, 2019). By delivering imperceptible, zero-mean stochastic electrical currents to the bilateral mastoid processes, nGVS enhances sensory perception of vestibular inputs *via* the principle of stochastic resonance (McLaren et al., 2023). Rather than directly correcting peripheral vestibular pathology, sub-threshold stochastic noise lowers the perception threshold of primary afferent neurons, thereby increasing their signaling sensitivity and temporal precision (Mitsutake & Sonobe, 2024; Wuehr et al., 2018). Our published study showed that nGVS optimized central vestibular signal processing and multisensory integration in healthy young adults, as evidenced by reduced dorsolateral prefrontal cortex activation alongside increased supramarginal gyrus engagement (Xie et al., 2026). Increasing clinical evidence further indicates that nGVS-enhanced vestibular feedback improves dynamic postural control and gait performance in populations with sensorimotor impairments, such as vestibulopathy (Eder et al., 2022; Fujimoto et al., 2018; Wuehr et al., 2023), supranuclear palsy (Wuehr et al., 2024), and Parkinson’s disease (Tran et al., 2023), underscoring its therapeutic potential. Crucially, Cyr et al. (2025) observed that scoliotic adolescents exhibited significantly greater gains in postural control, reflected by enhanced symmetry in vestibular-evoked balance responses, under nGVS compared to healthy controls. Our previous work further confirmed that nGVS significantly reduced medial-lateral GRF peaks and impulses in patients with AIS during level walking, demonstrating its efficacy and safety in modulating gait kinetics (Xie et al., 2025). However, kinetic metrics only provide a global representation of weight transfer, a mechanistic understanding of nGVS effects requires the integration of both kinematics and kinetics (Fortin, Nadeau & Labelle, 2008). Given that scoliosis compromises multi-segmental motor control (*e.g.*, trunk, pelvis, and lower limbs), Garg et al. (2021) highlighted the necessity of examining kinematic alterations across the spine-pelvis-hip complex when evaluating gait in AIS. Nevertheless, the multi-segmental biomechanical mechanisms through which nGVS modulates gait symmetry in AIS remain unclear. Furthermore, existing gait analyses for AIS have predominantly focused on level walking, overlooking the heightened biomechanical demands imposed by challenging locomotor tasks (*e.g.*, stair negotiation) (Gou et al., 2024a). As demonstrated by Wang, Chien & He (2025), stair negotiation provides a greater sensorimotor challenge than level walking, which may amplify subtle gait asymmetries. Whether nGVS exerts task-specific effects on gait symmetry across diverse locomotor conditions warrants investigation.

Consequently, this study aims to investigate the effects of the nGVS on medial-lateral gait symmetry during level walking and single-step stair negotiation in patients with AIS. It was hypothesized that (1) nGVS would enhance gait symmetry through improved SI and Xcorr for spine-pelvis-hip kinematics and kinetics; (2) effects of nGVS would differ between level walking and stair negotiation; and (3) nGVS-induced modulation of gait symmetry would correlate with spinal deformity severity. By concurrently analyzing kinetic and kinematic metrics, this study would provide a comprehensive biomechanical profile to characterize the neuromodulatory effects of nGVS on gait symmetry in AIS, particularly under varying task demands and deformity severities.

**Figure 1:**
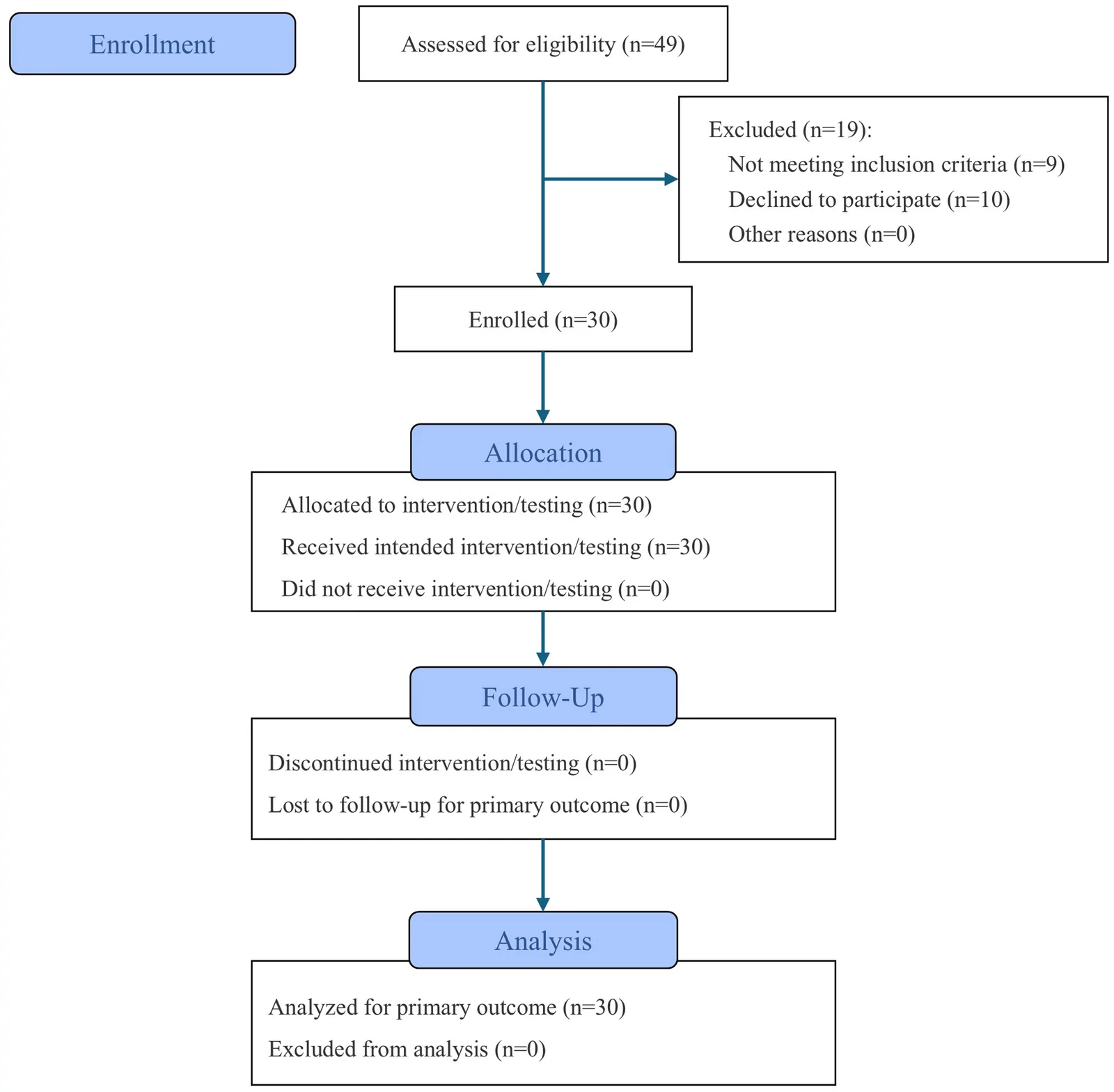
CONSORT flow diagram of this study. The flow diagram outlines the participant progression through recruitment, eligibility assessment, intervention/testing, data collection, and data analysis Flow diagram outlines the participant progression through assessing for eligibility, enrollment, intervention/testing, and data analysis in accordance with the CONSORT guidelines.

## Materials & Methods

### Participants

This single-group crossover study screened a total of 49 patients with AIS recruited from Suzhou Municipal Hospital *via* poster advertisements and clinical referrals between May 1 to June 5, 2026. Based on the inclusion/exclusion criteria, 30 eligible participants were enrolled in this study. The detailed study flow was presented in Fig. 1. The inclusion criteria were as follows: (1) newly diagnosed, treatment-naive AIS confirmed by standing posteroanterior full-spine radiograph obtained on the day of data collection or within the preceding 3 days; (2) a primary Cobb angle between 10° and 30°; and (3) age between 10 and 17 years. Exclusion criteria comprised any significant musculoskeletal disorders, history of spinal injury or surgery that could influence posture and gait, or any vestibular impairments. Scoliosis-related radiographic parameters were quantified from the standing posteroanterior full-spine radiographs by an experienced radiologist. The demographic characteristics of all participants were detailed in Table 1. Notably, in this cohort, nine participants exhibited C-shaped scoliosis (single primary curve), and 21 exhibited S-shaped scoliosis (primary and secondary curves). For participants with S-shaped scoliosis, the secondary curve represented a minor compensatory adjustment (Farshad, Catanzaro & Schmid, 2016). Consequently, for each participant, the convex and concave sides across subsequent biomechanical analyses were strictly defined based on the lateral orientation of primary curves. The study was approved by the Ethics Committee of Suzhou Municipal Hospital (IRB# K-2026-010-K01), and had been enrolled in the Chinese Clinical Trial Registry (ChiCTR2600122747). Data collection commenced only after written informed consent was obtained from all participants and their guardians. This cohort was independent of our previous study populations, with no overlap in participant recruitment or data collection (Xie et al., 2025).

**Table 1:** Demographic characteristics of participants with adolescent idiopathic scoliosis (*n* = 30) in this study.

Characteristics		Statistics
Sex (male/female)	8/22	
Age (years)	13.02 ± 2.08	
Height (cm)	160.98 ± 10.85	
Weight (kg)	44.83 ± 10.75	
Body mass index (kg/m^2^)	17.14 ± 3.02	
Types of scoliosis (*n*)		
C-shaped	9	
S-shaped	21	
Cobb angle of primary curve (°)		
C-shaped	18.10 ± 6.38	*t*= 0.813, *p*= 0.423
S-shaped	19.76 ± 4.41	
Total (*n* = 30)	18.60 ± 5.84	
Cobb angle of secondary curve (°)		
C-shaped	/	
S-shaped	12.13 ± 1.55	
Orientation of primary curve (*n*)		
Left	16	*χ*^2^ = 0.133, *p*= 0.715
Right	14	
Vestibular perception threshold (mA)	1.36 ± 0.44	

**Notes.**

Data were presented as mean ± standard deviation for continuous variables and ratios for categorical variables. *p* > 0.05 indicated no statistically significant difference in Cobb angle magnitudes between S-shaped and C-shaped primary curves, and no statistically significant difference in participant distribution between left- and right-sided primary curves.

To evaluate the statistical adequacy of our sample size for the within-subject design, a sensitivity power analysis was performed using G*Power software (version 3.1.9.4) for a two-way repeated-measures analysis of variance (ANOVA). With a significance level (*α*) of 0.05, statistical power (1 − β) of 0.80, number of measurements of 9, correlation among repeated-measures of 0.5, and nonsphericity correction of 1, the sensitivity power analysis revealed that the sample size of 30 was sufficiently powered to detect minimum within-subject main effects and interaction effect sizes as small as 0.028 *via* the *partial eta squared* (${\eta }_{p}^{2}$) method.

### Experimental setups

Following established protocols, nGVS was delivered as a zero-mean Gaussian white noise current *via* a portable electrical stimulator (BC102-IV; BrainCLOS Co., Ltd., Shenzhen, China) (Xie et al., 2026). A pair of circular silicone gel electrodes (surface area: 1.75 cm^2^) were securely positioned over the bilateral mastoid processes using hypoallergenic tape. Although previous nGVS studies predominantly adopt a broader bandwidth of 0–30 Hz to approximate the natural frequency range of the vestibular system for hair cell activation, the frequency of nGVS in this study was band-limited to 4–20 Hz based on our prior work demonstrating its efficacy in improving gait kinetics and dynamic stability in AIS (McLaren et al., 2023; Xie et al., 2025). Because the nGVS profile was constrained to a zero mean, its instantaneous amplitude fluctuated stochastically around 0 mA, producing dynamic polarity reversals across the binaural electrodes while eliminating net direct-current charge accumulation (McLaren et al., 2023). Individual stimulation intensity was set at 80% of each participant’s vestibular perception threshold (VPT), a level previously identified as optimal sub-threshold amplitude for healthy cohorts (Wuehr et al., 2018). The VPT was pre-determined during quiet standing using a stepwise ascending/descending procedure consistent with our prior protocol (Xie et al., 2026). For each nGVS trial, stimulation onset was synchronized with gait initiation and maintained throughout task execution (approximately 5–7 s per trial), ceasing immediately upon trial completion. Due to its sub-threshold nature, nGVS remained imperceptible to participants, preventing localized cutaneous sensation or startle responses upon onset. In the sham-stimulation condition, the nGVS device was identically fitted but delivered no current (0 mA) to mitigate the placebo effect, whereas no device was worn in the no-stimulation condition.

Kinematic and kinetic data were acquired in a time-synchronized manner. Three-dimensional kinematics were captured at 120 Hz using a motion capture system (Vicon Motion Systems Ltd., Oxford, UK) equipped with eight high-resolution infrared cameras (Vero 2.2). Following the guidelines of the International Society of Biomechanics, a gait model was established based on seventeen 15-mm spherical retro-reflective markers affixed to following anatomical landmarks: the spinal process of vertebra prominens (C7), acromion, anterior and posterior superior iliac spine (ASIS, PSIS), greater trochanter (GT), lateral epicondyle of the femur (LE), lateral malleoli (LM), second metatarsophalangeal ray (toe), and calcaneal tuberosity (heel) (Fig. 2A) (Wu et al., 2002; Xie et al., 2024). Simultaneously, GRF data were collected at 1,000 Hz using a force plate (BMS400600; AMTI, Watertown, USA) embedded midway along the walkway.

**Figure 2:**
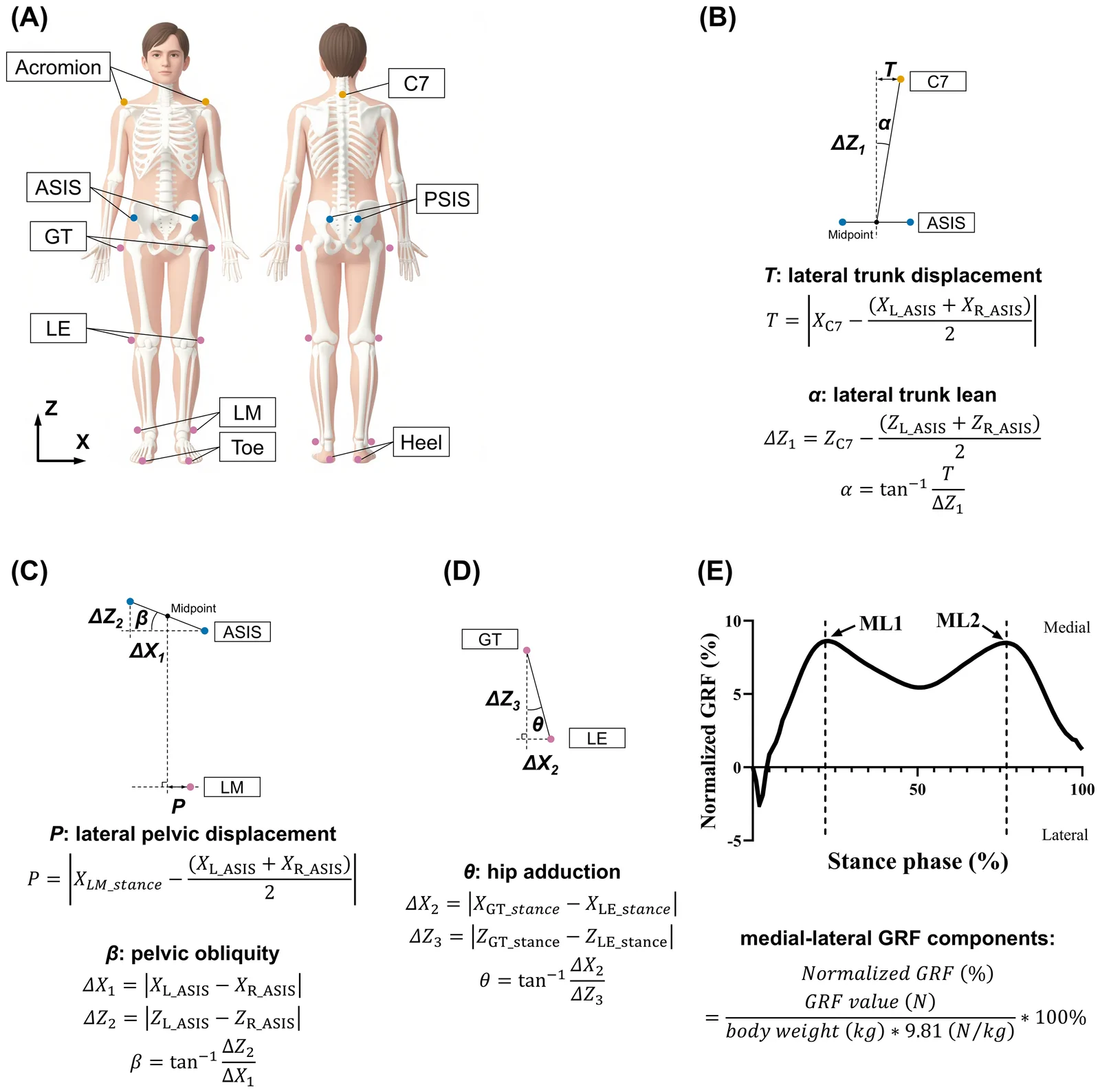
Marker placement and definition of gait kinematic and kinetic measures. (A) The location of 17 markers from front and back views. (B–D) Definition of gait kinematic measures of trunk, pelvis, and hip in the frontal plane. A geometrical model was used to extract analytical expression for each gait kinematic variable. (E) The selected ground reaction force (GRF) peaks in the medial-lateral directions. ASIS, anterior superior iliac spine; C7, the spinal process of vertebra prominens; GT, greater trochanter; LE, lateral epicondyle; LM, lateral malleoli; ML1, first peak in the medial-lateral direction; ML2, second peak in the medial-lateral direction; PSIS, posterior superior iliac spine.

### Experimental protocols

A custom-built, non-slip, single-step staircase with a riser height of 150 mm was used for stair negotiation in this study (Fig. 3A). This staircase complied with the specification for public building stairs as outlined in the Chinese General Code for Civil Buildings (GB 55031-2022) and International Building Code (IBC 1011.5.2).

**Figure 3:**
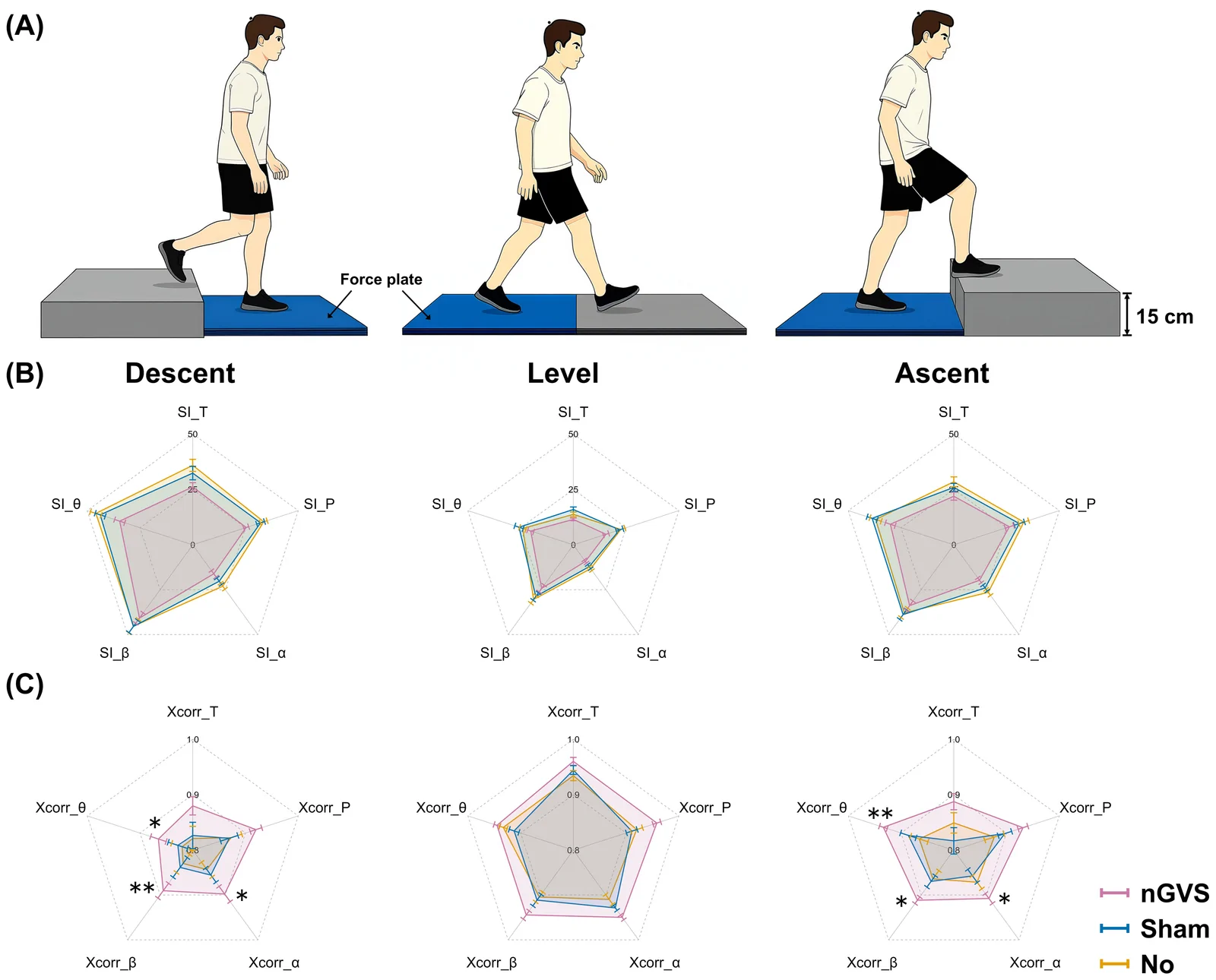
Experimental protocols and results of gait symmetry for kinematic parameters. (A) Illustration of the tasks in this study, including level walking (Level) and single-step stair negotiation (Descent and Ascent). (B–C) Radar plots of gait symmetry for kinematic parameters across different tasks: (B) symmetry index (SI) and (C) normalized cross-correlation coefficient (Xcorr). Solid lines represent group means, and error bars indicate ±1 standard error of the mean (SEM). nGVS, noisy galvanic vestibular stimulation; No, no stimulation; P, lateral pelvic displacement; Sham, sham stimulation; T, lateral trunk displacement; *α*, lateral trunk lean angle; *β*, pelvic obliquity angle; *θ*, hip adduction angle. *, *p* < 0.05; **, *p* < 0.01.

Prior to data collection, key anthropometric characteristics (*e.g.*, weights and heights) of participants were obtained. Subsequently, participants performed three types of tasks (level walking, stair ascent, and stair descent) at a consistent, self-selected speed across three stimulation conditions (nGVS, sham-stimulation, and no-stimulation). For each task, separate trials were conducted where participants were explicitly instructed to lead with either their convex- or concave-side foot when stepping onto the force plate. This protocol yielded a total of 18 experimental conditions (3 tasks × 3 stimulation conditions × 2 leading feet) per participant, with each condition repeated three times. All trials were executed in a fully randomized order. A trial was defined as successful if the participant completed locomotor tasks smoothly without hesitation, stumbling, loss of balance, handrail reliance, or significant marker occlusion. Unsuccessful trials were discarded immediately and repeated after a brief rest period until three successful trials per condition were recorded. For each trial, the start line was set two steps away from the force plate to allow for natural gait adjustments (Chai et al., 2023). Following stair negotiation, participants were instructed to continue walking for three additional steps beyond the force plate to a designated finish line. For level walking, participants walked from the start to finish line, covering a distance of approximately six steps, ensuring that the third step would naturally land on the force plate. Participants were instructed to perform all tasks within their limits of safety and were permitted to rest between trials if necessary.

### Data analysis

#### Processing of kinetic and kinematic data

Data processing was conducted in MATLAB 2023b (MathWorks, Natick, USA). For kinematic analysis, raw marker trajectories were smoothed using a zero-lag fourth-order low-pass Butterworth filter with a cut-off frequency of six Hz (Wang, Xie & Chien, 2024a; Yang et al., 2013). For kinetic analysis, the voltage output from each force plate channel was converted to force (in Newtons) by applying the respective calibration factors. Subsequently, the resulting GRF data were processed with a zero-lag fourth-order low-pass Butterworth filter with a 10 Hz cut-off frequency (Wang, Xie & Chien, 2024b; Xie et al., 2025). These unified filters for kinematic and kinetic data effectively mitigated high-frequency motion artifacts and system noise while fully preserving trajectory morphology and temporal dynamics for waveform-based analysis (Sinclair, Taylor & Hobbs, 2013).

#### Kinematic and kinetic parameter extraction

For each experimental condition, three successful trials with the convex side or concave side as the leading foot were retained and averaged to generate kinematic and kinetic profiles for subsequent data analysis. For kinematic analysis, the peak values of the medial-lateral displacements and angles of the trunk, pelvis, and hip in the frontal plane were extracted during the stance phase, defined as the interval between initial contact and toe-off. These critical gait events were identified based on the vertical GRF, in accordance with our previous study (Xie et al., 2024). Specifically, initial contact was defined as the first time point at which the vertical GRF exceeded 10 N and remained above this threshold for 40 ms; similarly, toe-off was defined as the time point at which the vertical GRF fell below 10 N and sustained for 40 ms. Then, all data trajectories were time-normalized from 0% to 100% of the same stance phase. The key kinematic parameters were calculated as follows: (1) lateral trunk displacement (T) and lateral trunk lean angle (*α*): derived from the position and orientation of the C7 marker relative to the pelvic center (midpoint of the bilateral ASIS markers) (Tokuda et al., 2018). This trunk-pelvis coordinate frame decoupled active spine-on-pelvis motion from downstream lower-limb movement and whole-body spatial translation (Gerbrands et al., 2017; Sheng et al., 2025); (2) lateral pelvic displacement (P): calculated as the relative medial-lateral offset between the pelvic center and the LM marker of the stance foot, utilizing a center-of-mass to base-of-support spatial framework (Dodd & Morris, 2003; Lu, Xie & Chien, 2022). Based on the dynamic margin of stability principle, this calculation isolated pelvic displacement relative to the supporting base while eliminating confounding artifacts of swing-limb trajectories (Hof, Gazendam & Sinke, 2005; Wang, Xie & Chien, 2024a); (3) pelvic obliquity (*β*): defined as the frontal-plane angular inclination of the line connecting the bilateral ASIS markers relative to the global horizontal axis (Dodd & Morris, 2003); and (4) the hip adduction angle (*θ*): calculated by the spatial relationship between the ipsilateral GT and LE markers of the stance foot (Yang et al., 2013). All specific calculations were performed according to the schematic and formulae provided in Figs. 2B–2D. For kinetic analysis, the filtered GRF data in the medial-lateral direction were normalized to individual body weights to ensure the comparability across participants. Subsequently, two peak values from medial-lateral GRF components were extracted: the first peak during the braking phase (ML1) and the second peak during the pushing-off phase (ML2) (Fig. 2E).

#### Static offset correction for kinematic data

To isolate dynamic postural adaptations from fixed structural deformities inherent to AIS (*e.g.*, rigid spinal curvature, pelvic asymmetry, and altered trunk alignment), kinematic waveforms across all conditions were adjusted by subtracting each participant’s static offset measured during quiet stance prior to data collection (Schwartz, Rozumalski & Trost, 2008; Wu et al., 2019). According to Dalleau et al. (2012), inherent structural deformities imposed a baseline geometric bias that may amplify or mask dynamic movement ranges during locomotion. A similar biomechanical rationale was established in a knee osteoarthritis study, where the osteoarthritis-related alignment offsets measured from a static trial significantly confounded dynamic knee kinetics during gait (Althomali, 2024). Consequently, zero-referencing each participant to their quiet standing baseline prevented static structural noise from confounding condition-wise comparisons. Mathematically, this correction represented an additive constant baseline shift. Because the mean-centering procedure was incorporated into the calculation of waveform similarity (Xcorr), Xcorr remained strictly invariant to this baseline translation (as detailed below). Conversely, magnitude-based metrics (SI) were directly altered by baseline subtraction. Therefore, to confirm that static offset correction did not introduce analytical bias or mask effects on SI, a sensitivity analysis using uncorrected kinematic data was performed.

#### Calculation of gait symmetry

Prior to symmetry analysis, all kinematic and kinetic waveforms were directionally normalized to a unified reference frame. As the global coordinate system remained fixed with the medial-lateral axis (*X*-axis) defined as positive toward the right, raw trajectories from the convex and concave sides naturally exhibited opposing algebraic signs. To establish a standardized reference frame, kinematic and kinetic trajectories across all trials were directionally normalized to a uniform positive direction. This directional normalization ensured equivalent spatial comparison while fully preserving the original trajectory morphology and temporal dynamics of all parameters.

Building upon the data processing described above, two complementary metrics were employed to evaluate gait symmetry using customized MATLAB scripts. First, the SI was calculated to quantify the asymmetry with following formula:

\begin{eqnarray*}SI=2\ast \left\vert \frac{{X}_{Convex}-{X}_{Concave}}{{X}_{Convex}+{X}_{Concave}} \right\vert \ast 100\% \end{eqnarray*}

where *X*_*Convex*_ and *X*_*Concave*_ denote the averaged peak values obtained from trials where participants led with their convex-side and concave-side limbs, respectively. A higher SI approaching 100% indicated greater asymmetry, while 0% reflects perfect symmetry. Absolute values prevent positive and negative deviations from cancelling out, thereby avoiding masking of true symmetry (Xie et al., 2024).

Second, the zero-mean Xcorr was used to assess time-series waveform similarity between concave and convex sides. The time-normalized waveforms across bilateral sides, spanning 0–100% of each stance phase, were discretized into *N* = 101 points and denoted as *Y*_*Convex*_*(n)* and *Y*_*Concave*_*(n)*, respectively. To eliminate the influence of constant baseline shifts, dynamic mean-centering was applied across the stance phase: ${\tilde {Y}}_{Convex} \left( n \right) ={Y}_{Convex} \left( n \right) -{\overline{Y}}_{Convex}$ and ${\tilde {Y}}_{Concave} \left( n \right) ={Y}_{Concave} \left( n \right) -{\overline{Y}}_{Concave}$, where $\overline{Y}$ represents the temporal mean of the waveform. Xcorr was then computed using the mean-centered waveforms as follows (Ogihara et al., 2020):

\begin{eqnarray*}CC(k)& =\sum _{n=1}^{N}{\tilde {Y}}_{Convex} \left( n \right) {\tilde {Y}}_{Concave}{ \left( n-k \right) }_{\mathrm{if}n-k\leq 0\mathrm{or}n-k> N\mathrm{then}{\tilde {Y}}_{Concave} \left( n-k \right) =0}^{k=0,\pm 1,\pm 2,\ldots \pm N-1} \end{eqnarray*}

\begin{eqnarray*}Ac{Y}_{Convex}(k)& =\sum _{n=1}^{N}{\tilde {Y}}_{Convex} \left( n \right) {\tilde {Y}}_{Convex}{ \left( n-k \right) }_{\mathrm{if}n-k\leq 0\mathrm{or}n-k> N\mathrm{then}{\tilde {Y}}_{Convex} \left( n-k \right) =0}^{k=0,\pm 1,\pm 2,\ldots \pm N-1} \end{eqnarray*}

\begin{eqnarray*}Ac{Y}_{Concave}(k)& =\sum _{n=1}^{N}{\tilde {Y}}_{Concave} \left( n \right) {\tilde {Y}}_{Concave}{ \left( n-k \right) }_{\mathrm{if}n-k\leq 0\mathrm{or}n-k> N\mathrm{then}{\tilde {Y}}_{Concave} \left( n-k \right) =0}^{k=0,\pm 1,\pm 2,\ldots \pm N-1} \end{eqnarray*}

\begin{eqnarray*}Xcorr& = \frac{\max \nolimits (CC(k))}{\sqrt{Ac{Y}_{Convex} \left( 0 \right) Ac{Y}_{Concave}(0)}} \end{eqnarray*}

where *k* represents the time lag between bilateral waveforms. In the present study, Xcorr was evaluated at the optimal time lag that yielded the maximum waveform similarity, *max(CC(k))*. This approach intentionally compensated for minor inter-limb temporal phase desynchronizations or internal event-detection timing offsets, thereby isolating spatial waveform similarity from temporal phase shifts (Ogihara et al., 2020; Wang, Chien & He, 2025). Moreover, as the mean-centering procedure algebraically canceled out the additive constant offset, the calculated Xcorr was strictly invariant to static offset corrections. Xcorr ranged from 0 to 1, where a value close to 1 indicated high waveform similarity (high symmetry) and a value approaching 0 indicated low waveform similarity (low symmetry).

#### Statistical analysis

In accordance with our clinical trial registration, the aforementioned kinematic and kinetic parameters, along with their corresponding symmetry metrics, were all registered as outcome parameters. However, to directly test the hypotheses of this study, which explicitly focused on medial-lateral gait symmetry, outcome measures were prioritized as follows: (1) primary outcomes: SI and Xcorr across kinematic and kinetic parameters; and (2) secondary outcomes: peak values of kinematic and kinetic parameters. Notably, to maintain a clear analytical focus on gait symmetry while ensuring complete data transparency, secondary outcomes were summarized in the Supplemental Information without formal statistical analysis.

All statistical analyses were conducted using SPSS 27.0 (IBM Corporation, Armonk, USA). The normality of the data for all variables was assessed using the *Shapiro–Wilk* test. For normally distributed data, a two-way repeated-measures ANOVA with two within-subject factors (stimulation conditions: nGVS/sham/no; tasks: level walking/stair descent/stair ascent) was employed to examine main effects and interactions of each primary outcome. The assumption of sphericity was assessed using *Mauchly’s* test, and degrees-of-freedom were adjusted using the *Greenhouse-Geisser* correction when violated. For non-normally distributed data, the *Friedman* test was applied. To control the family-wise error rate across factor levels, *post-hoc* pairwise comparisons were conducted using *Bonferroni*-corrected tests (or *Wilcoxon signed-rank* tests with *Bonferroni*-correction for non-parametric data). Given the three stimulation conditions, *post-hoc* comparisons were hierarchically structured: the (nGVS *vs.* sham) contrast was designated as the primary analysis of true neuromodulatory efficacy (controlling for placebo and device-related factors), whereas the (nGVS *vs.* no) contrast served as the secondary analysis. All interpretations of nGVS effects were strictly based on the (nGVS *vs.* sham) contrast. Effect sizes for interaction and main effects were quantified using ${\eta }_{p}^{2}$, interpreted according to Cohen’s guidelines (large: ≥0.138; medium: ≥0.058; small: ≥0.009) (Cohen, 1988). Furthermore, empirical ${\eta }_{p}^{2}$ values were compared against the pre-determined sensitivity threshold (${\eta }_{p}^{2}=0.028$) to confirm that all primary outcome measures achieved adequate statistical power.

To test whether nGVS efficacy was modulated by spinal deformity severity while controlling for multiplicity across outcomes, a conditional targeted correlation analysis was performed exclusively for variables that demonstrated either a significant two-way interaction or a significant main effect of stimulation conditions (Ma et al., 2020). To isolate the specific neuromodulatory effect of nGVS from non-specific effects (*e.g.*, placebo, device-related factors), a sham-controlled net efficacy index (Δ) was calculated for each symmetry metric as:

\begin{eqnarray*}\Delta ={X}_{nGVS}-{X}_{Sham} \end{eqnarray*}

where *X*_*nGVS*_ and *X*_*Sham*_ denote the values under the nGVS and sham-stimulation conditions, respectively. Relationships between Cobb angle and Δ were examined using *Pearson* or *Spearman’s* rank correlation coefficients, with the choice determined by data normality. The analytical approach followed two pathways: (1) if a significant stimulation × task interaction was observed, correlations were computed separately for each task; (2) if only a significant main effect of stimulation was present (without significant interaction), Δ values were averaged across the three tasks prior to correlation analysis. Correlation strength was interpreted based on Cohen’s guidelines (large: |*r*| ≥ 0.50; medium: |*r*| ≥ 0.30; small: |*r*| ≥ 0.10) (Cohen, 1988). The significant level was set at 0.05.

## Results

All participants successfully completed the experimental protocol without any adverse events, loss of balance, or trial failures; thus, no additional trials were performed. For all dependent variables evaluated in the two-way repeated-measures ANOVA, the empirical effect sizes for all interaction and main effects (${\eta }_{p}^{2}=0.033\sim 0.572$, detailed in Table 2) consistently exceeded the sensitivity threshold (${\eta }_{p}^{2}=0.028$), confirming that the sample size of 30 provided adequate statistical power. Detailed data for kinematic/kinetic parameters and respective symmetry measures are summarized in Tables S1–S2. To illustrate the temporal alterations across the stance phase, representative time-series trajectories of frontal-plane kinematic and kinetic parameters from a typical participant are shown in Fig. 4.

**Table 2:** Summary of statistical results of symmetry metrics from the two-way repeated-measures ANOVA.

	Effect	*F*	*df*_*effect*_, *df*_*error*_	*p*	${\eta }_{p}^{2}$
SI_ML1 (%)	Interaction	3.451	3.28, 95.12	0.011^*^	0.106
	Stimulation	5.943	1.32, 38.26	0.004^**^	0.170
	Task	2.682	1.86, 53.94	0.077	0.085
SI_ML2 (%)	Interaction	1.789	3.32, 96.28	0.133	0.058
	Stimulation	3.108	1.69, 49.01	0.052	0.097
	Task	2.746	1.88, 54.54	0.073	0.086
SI_T (%)	Interaction	0.986	4, 116	0.417	0.033
	Stimulation	1.203	2, 58	0.308	0.040
	Task	2.300	2, 58	0.109	0.073
SI_*α* (%)	Interaction	1.120	4, 106	0.350	0.037
	Stimulation	9.446	2, 58	<0.001^***^	0.246
	Task	3.019	2, 58	0.057	0.094
SI_P (%)	Interaction	1.983	4,116	0.099	0.064
	Stimulation	2.256	2, 58	0.114	0.072
	Task	1.779	2, 58	0.178	0.058
SI_*β* (%)	Interaction	2.018	4,116	0.094	0.065
	Stimulation	5.937	2, 58	0.005^**^	0.170
	Task	3.054	2, 58	0.055	0.095
SI_*θ* (%)	Interaction	1.738	2.98, 86.42	0.146	0.057
	Stimulation	0.980	1.93, 55.97	0.381	0.033
	Task	1.253	1.74, 50.47	0.293	0.041
Xcorr_ML	Interaction	7.813	3.44, 99.76	<0.001^***^	0.212
	Stimulation	28.942	1.70, 49.24	<0.001^***^	0.499
	Task	38.741	1.95, 56.54	<0.001^***^	0.572
Xcorr_T	Interaction	1.858	4, 116	0.122	0.060
	Stimulation	1.532	2, 58	0.225	0.050
	Task	2.107	2, 58	0.131	0.068
Xcorr_*α*	Interaction	5.003	3.60, 104.4	0.001^**^	0.147
	Stimulation	7.722	1.64, 47.40	0.001^**^	0.210
	Task	4.521	1.52, 43.95	0.015^*^	0.135
Xcorr_P	Interaction	2.221	3.48, 100.92	0.090	0.071
	Stimulation	1.780	1.62, 46.98	0.189	0.058
	Task	1.579	1.99, 57.91	0.214	0.051
Xcorr_*β*	Interaction	5.331	4,116	0.002^**^	0.155
	Stimulation	24.989	2, 58	<0.001^***^	0.463
	Task	1.322	2, 58	0.275	0.044
Xcorr_*θ*	Interaction	2.811	3.06, 88.73	0.029^*^	0.088
	Stimulation	1.560	1.87, 54.27	0.219	0.051
	Task	1.256	1.87, 54.26	0.267	0.041

**Notes.**

*df* degrees of freedom ML1 first ground reaction force (GRF) peak in the medial-lateral (ML) direction ML2 second GRF peak in the ML direction P lateral pelvic displacement SI symmetry index T lateral trunk displacement Xcorr normalized cross-correlation coefficients *α* lateral trunk lean *β* pelvic obliquity *θ* hip adduction

* *p* < 0.05.

** *p* < 0.01.

*** *p* < 0.001.

**Figure 4:**
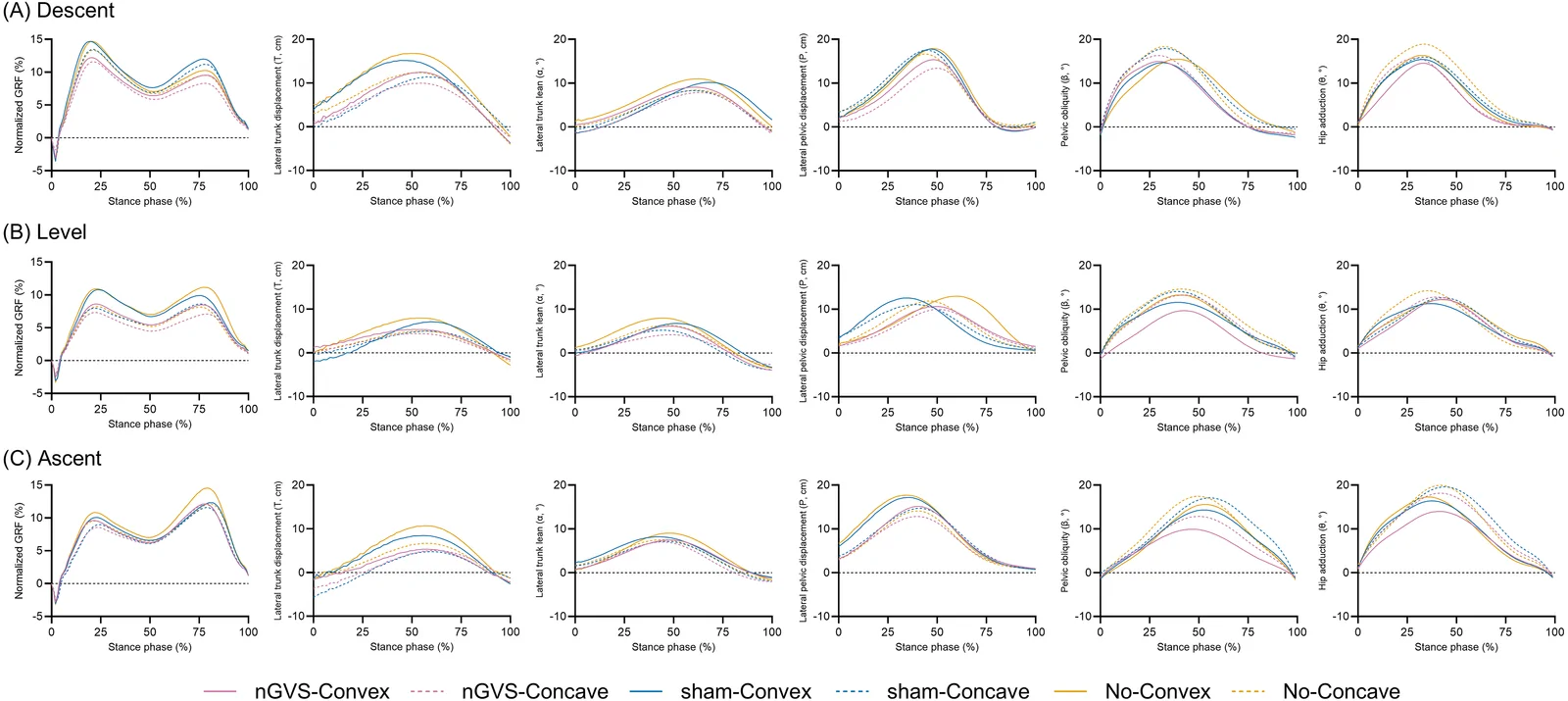
Representative time-series trajectories of medial-lateral kinematic and kinetic parameters across the stance phase during different locomotor tasks. Time-series waveforms derived from a representative participant are plotted from 0% to 100% of the stance phase during (A) stair descent, (B) level walking, and (C) stair ascent. Concave, trials leading with the concave-side limb; Convex, trials leading with the convex-side limb; nGVS, noisy galvanic vestibular stimulation; No, no stimulation; Sham, sham stimulation.

### Effects of nGVS on symmetry metrics

As presented in Table 2, significant two-way interactions were observed follow following kinematic symmetry metrics: Xcorr_α (*F*_3.60,104.42_ = 5.003, *p* = 0.001, ${\eta }_{p}^{2}=0.147$), Xcorr_β (*F*_4,116_ = 5.331, *p* = 0.002, ${\eta }_{p}^{2}=0.155$), and Xcorr_θ (*F*_3.06,88.73_ = 2.811, *p* = 0.029, ${\eta }_{p}^{2}=0.088$). *Post-hoc* comparisons prioritizing the (nGVS *vs.* sham) contrast revealed that:(1) during stair descent, nGVS significantly enhanced Xcorr_α (*p* = 0.017) and Xcorr_β (*p* = 0.006) compared to sham-stimulation; (2) during stair ascent, nGVS significantly increased Xcorr_α (*p* = 0.033) and Xcorr_θ (*p* = 0.008) relative to sham-stimulation (Figs. 3B–3C). These significant comparisons were also evident in the (nGVS *vs.* no) contrast (all *p* < 0.05). Additionally, compared to no stimulation, nGVS significantly increased Xcorr_θ during descent (*p* = 0.030) and Xcorr_β during ascent (*p* = 0.021). Although there was no significant two-way interaction, significant main effects of stimulation condition were observed for SI_α (*F*_2,58_ = 9.446, *p* < 0.001, ${\eta }_{p}^{2}=0.246$) and SI_β (*F*_2,58_ = 5.937, *p* = 0.005, ${\eta }_{p}^{2}=0.170$), with nGVS significantly reducing both metrics relative to sham- and no-stimulation (all *p* < 0.05). A sensitivity analysis using kinematic data without static offset correction confirmed the robust neuromodulatory effects of nGVS on SI of kinematic parameters. Specifically, the main effect of stimulation condition on SI_α remained statistically significant (*F*_2,58_ = 5.309, *p* = 0.008, ${\eta }_{p}^{2}=0.155$) based on the uncorrected dataset. In contrast, for SI_β, uncorrected kinematic data demonstrated increased inter-subject variance and violated the assumption of sphericity, as evidenced by non-significant main effects of stimulation condition (*F*_1.58,42.82_ = 3.803, *p* = 0.058, ${\eta }_{p}^{2}=0.123$) after the degrees-of-freedom adjustment (Table S3). The above-described findings confirmed that static offset correction removed static structural noise, restored statistical sphericity, and enhanced analytical sensitivity to detect the neuromodulatory effect of nGVS.

For kinetic symmetry metrics, significant two-way interactions were observed for SI_ML1 (*F*_3.28,95.12_ = 3.451, *p* = 0.011, ${\eta }_{p}^{2}=0.106$) and Xcorr_ML (*F*_3.44,99.76_ = 7.813, *p* < 0.001, ${\eta }_{p}^{2}=0.212$) (Fig. 5). *Post-hoc* comparisons prioritizing the (nGVS *vs.* sham) contrast revealed that: (1) nGVS significantly reduced SI_ML1 during descent (*p* = 0.015) and ascent (*p* = 0.022); (2) nGVS significantly enhanced Xcorr_ML during stair descent (*p* < 0.001).

**Figure 5:**
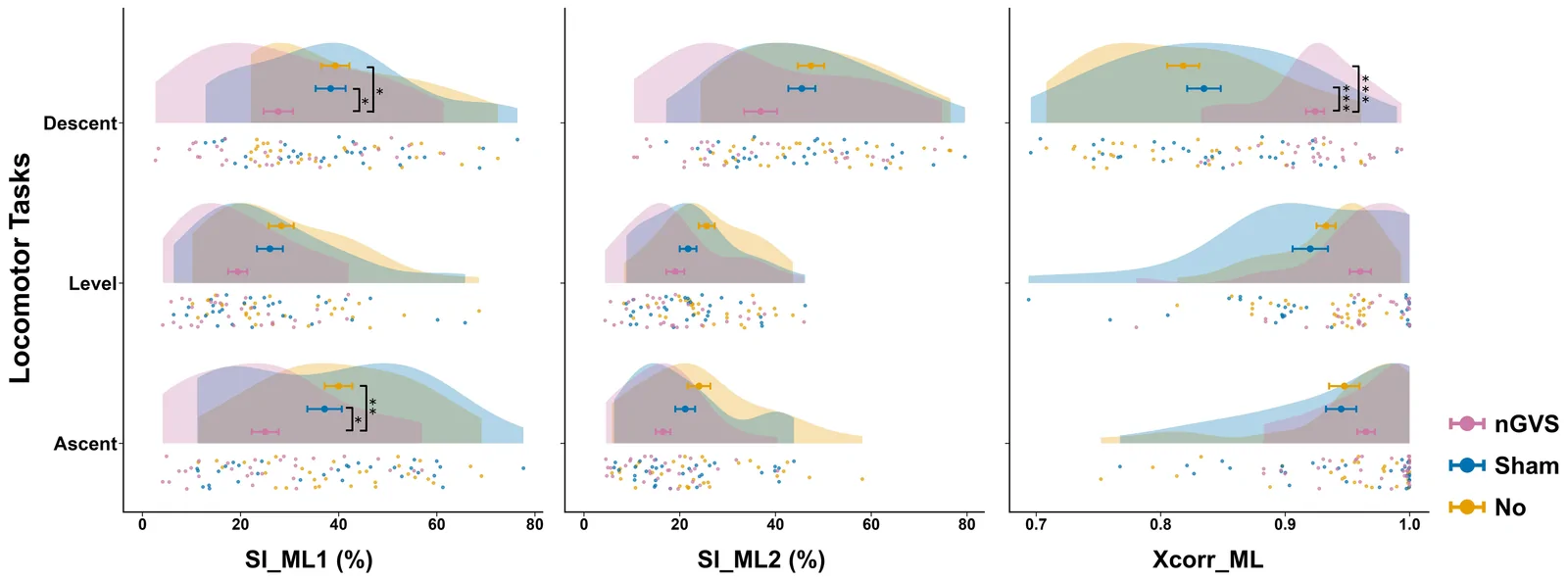
Raincloud plots of gait symmetry for kinetic parameters across different locomotor tasks:symmetry index (SI) and normalized cross-correlation coefficient (Xcorr). ML1, first ground reaction force (GRF) peak in the medial-lateral (ML) direction; ML2, second GRF peak in the ML direction; nGVS, noisy galvanic vestibular stimulation; No, no stimulation; Sham, sham stimulation. *, *p* < 0.05; **, *p* < 0.01; ***, *p* < 0.001.

### Correlations of nGVS-induced symmetry improvements and spinal deformity severity

For variables exhibiting a significant two-way interaction, correlation analyses revealed a state-dependent pattern that nGVS-induced improvements of gait symmetry were correlated with spinal deformity severity (Table 3). Specifically, during stair descent, Cobb angle demonstrated significant positive correlations with Δ Xcorr_ML (*ρ* = 0.583, *p* = 0.001), Δ Xcorr_α (*r* = 0.539, *p* = 0.002), and Δ Xcorr_β (*ρ* = 0.394, *p* = 0.031), while it was negatively correlated with Δ SI_ML1 (*ρ* = −0.444, *p* = 0.014). During stair ascent, Cobb angle exhibited significant positive correlations with Δ Xcorr_ML (*r* = 0.575, *p* = 0.001), Δ Xcorr_α (*ρ* = 0.427, *p* = 0.019), and Δ Xcorr_θ (*r* = 0.398, *p* = 0.030). For SI_α and SI_β, which showed only significant main effects of stimulation condition, correlation analysis based on their averaged Δ values across three tasks revealed no significant associations with Cobb angle (Δ SI_α: *r* =  − 0.394, *p* = 0.137; Δ SI_β: *r* =  − 0.423, *p* = 0.108).

**Table 3:** Summary of correlations between Cobb angle and sham-controlled net efficacy (Δ) of noisy galvanic vestibular stimulation.

Symmetry metrics	Tasks			Averaged across tasks
	Stair descent	Level walking	Stair ascent	
Δ SI_ML1	−0.444 (0.014)^*^	0.239 (0.203)	0.051 (0.787)	/
Δ SI_*α* (%)	/	/	/	−0.394 (0.137)
Δ SI_*β* (%)	/	/	/	−0.423 (0.108)
Δ Xcorr_ML	0.583 (0.001)^**^	0.142 (0.453)	0.575 (0.001)^**^	/
Δ Xcorr_*α*	0.539 (0.002)^**^	0.049 (0.797)	0.427 (0.019)^*^	/
Δ Xcorr_*β*	0.394 (0.031)^*^	0.044 (0.817)	0.035 (0.855)	/
Δ Xcorr_*θ*	0.289 (0.121)	0.224 (0.233)	0.398 (0.030)^*^	/

**Notes.**

Data were presented as correlation coefficient (*p*-value).

ML1 first ground reaction force (GRF) peak in the medial-lateral (ML) direction SI symmetry index Xcorr normalized cross-correlation coefficient *α* lateral trunk lean *β* pelvic obliquity *θ* hip adduction

* *p* < 0.05.

** *p* < 0.01.

## Discussion

The present study demonstrated that nGVS improved medial-lateral gait symmetry in patients with AIS during single-step stair negotiation. Specifically, nGVS-induced improvements in medial-lateral gait symmetry were observed in SI and Xcorr metrics across both kinematic and kinetic domains. Notably, these biomechanical benefits derived from nGVS were task-specific, emerging exclusively during stair negotiation rather than level walking, and state-dependent, scaling with Cobb angles. Collectively, our findings revealed that nGVS ameliorated medial-lateral gait symmetry in AIS, with its efficacy contingent upon both task demands and spinal deformity severities.

### nGVS improved medial-lateral gait symmetry across kinematic and kinetic domains in AIS

In this study, nGVS significantly reduced asymmetry in lateral trunk lean, pelvic obliquity, and hip adduction, while synchronizing medial-lateral GRF patterns during stair negotiation in patients with AIS. These symmetry enhancements across individual kinematic segments within the spine-pelvis-hip complex, alongside global kinetic dynamics, indicated a synchronized refinement of multi-segmental spatial symmetry (Xie et al., 2024). Biomechanically, aberrant spine-pelvis alignment altered acetabular orientation and imposed asymmetric mechanical loading across bilateral lower limbs (Lafage et al., 2023). Therefore, stabilizing individual proximal segments (the spinopelvic complex) in the frontal plane was essential for establishing a symmetric foundation for distal movements (Zhang et al., 2021). In this context, application of nGVS led to reduced medial-lateral gait asymmetries in spinal and pelvic movements, as evidenced by significant increases in Xcorr_α and Xcorr_β, suggesting a more symmetric alignment of spinopelvic segments (Lafage et al., 2023). This proximal symmetry restoration subsequently accommodated more symmetric hip adduction kinematics, as reflected by the heightened Xcorr_θ under nGVS. Collectively, the nGVS-induced symmetry gains across individual kinematic segments within the spine-pelvis-hip complex illustrated a refinement of multi-segmental spatial symmetry, which underpinned the restoration of medial-lateral gait symmetry during single-step stair negotiation in AIS (Arauz et al., 2024).

Notably, kinetic symmetry metrics, particularly Xcorr_ML and SI_ML1, also demonstrated significant improvements during stair negotiation. As global integrators of whole-body dynamics, these alterations indicated a synchronized, symmetric medial-lateral kinetic profile under the application of nGVS (Mallau et al., 2007). While these nGVS-mediated symmetry improvements across gait kinematics and kinetics in AIS were unequivocal, underlying neurophysiological mechanisms remain inferential based on our biomechanical dataset. Gimmon et al. (2017) suggested that vestibular inputs served as an important source for modulating motor outputs that regulated dynamic posture and gait. Prior evidence also demonstrated the effectiveness of nGVS in improving gait stability in various populations (*e.g.*, patients with vestibulopathy and healthy adults) (Chen et al., 2021; Iwasaki et al., 2018; Wuehr et al., 2016a). It is therefore tentatively hypothesized that nGVS might optimize central vestibular signal processing to potentially compensate for vestibular dysfunction in AIS, thereby facilitating more symmetric motor execution. Nevertheless, in the absence of neuroimaging or electrophysiological assessments in this study, these biomechanical improvements should be interpreted with caution, as they represented indirect behavioral manifestations rather than neural evidence. Future investigations incorporating neuroimaging approaches are warranted to elucidate the precise neuromodulation mechanism engaged by nGVS.

### Task-specific responsiveness of gait symmetry modulation under nGVS

A critical finding of this study was the task-specific pattern of nGVS efficacy, with significant symmetry improvements emerging exclusively during stair negotiation, whereas no measurable enhancements were detected during level walking. From a biomechanical perspective, level walking was a highly automated locomotor task characterized by minimal center-of-mass displacement, brief single-limb support phases, and predictable balance control demands (Alsalaheen et al., 2014). Under these low-demand conditions, medial-lateral symmetry metrics, particularly Xcorr values, were predominantly maintained through habitual motor patterns and clustered near upper theoretical limits (typically Xcorr > 0.95) in patients with AIS. This high baseline gait symmetry induced a notable ceiling effect, constraining the statistical margin for detectable nGVS-induced improvements during low-demand level walking (Gouelle & Mégrot, 2016). Conversely, stair negotiation imposed substantially greater biomechanical demands due to increased medial-lateral center-of-mass excursions, prolonged single-limb support phases, and the necessity for precise multi-segmental control (Gou et al., 2024a). These heightened requirements shifted gait regulation from automatic execution toward feedback-driven processes reliant on postural adaptations (Wang, Chien & He, 2025). For patients with AIS, whose structural deformity inherently compromised baseline medial-lateral symmetry, the heightened biomechanical challenges of stair negotiation may place greater demands on dynamic postural control, potentially exposing subtle sensorimotor deficits and creating a functional window wherein nGVS could improve medial-lateral gait symmetry (Gou et al., 2024b; Gou et al., 2024c).

While both stair ascent and descent challenged medial-lateral gait symmetry, they imposed distinct biomechanical demands that elicited task-specific segmental control strategies in AIS. Our findings revealed that nGVS-induced symmetry improvements were localized to lateral trunk lean (*α*) and pelvic obliquity (*β*) during descent, whereas nGVS primarily enhanced hip adduction symmetry (*θ*) during ascent. This divergence underscored that nGVS did not uniformly alter symmetry across all segments, but rather accommodated control priorities of individual segments in accordance with task-specific biomechanical demands (Mackrous, Carriot & Cullen, 2022). During stair descent, the predominance of eccentric muscle actions and impact absorption necessitated robust stabilization of the spinopelvic complex to restrict excessive medial-lateral center-of-mass displacement and mitigate destabilizing perturbations (Riener, Rabuffetti & Frigo, 2002). In this context, the nGVS-induced improvements in Xcorr_α and Xcorr_β indicated enhanced spatial symmetry of the spinopelvic complex, contributing to trunk stabilization and reduced compensatory frontal-plane body sway (Wuehr et al., 2016b). In contrast, stair ascent was characterized by concentric propulsion, imposing substantial hip and knee extensor moments to elevate the center-of-mass while demanding precise hip control to maintain dynamic balance (Law et al., 2021). The nGVS-mediated symmetry improvements in hip adduction suggested that nGVS facilitated more symmetric medial-lateral hip control during the propulsive phase (Law et al., 2021). Collectively, these results demonstrated that nGVS operated through a task-specific pattern in AIS, selectively refining spatial gait symmetry across individual kinematic segments in accordance with the distinct biomechanical demands of varying locomotor tasks.

### State-dependent response to nGVS scaled with spinal deformity severity

To minimize spurious associations, conditional correlation analyses between spinal deformity severity and nGVS efficacy (Δ) were restricted to outcome measures exhibiting a significant interaction or main effect of stimulation conditions in the two-way repeated-measures ANOVA. This hypothesis-driven approach yielded a state-dependent relationship: the magnitude of nGVS-induced improvements in SI_ML1 and Xcorr_ML/α/β/θ correlated with the Cobb angle during stair negotiation, indicating that patients with more severe spinal deformities derived substantially greater kinematic and kinetic symmetry benefits from nGVS. This finding aligned with available evidence linking curve magnitude to gait asymmetry in AIS (Park et al., 2016). Assuming that patients with smaller Cobb angles presented with milder sensorimotor deficits, their baseline vestibular processing may operate near functional capacity (Vlădăreanu et al., 2024). This potentially induced a ceiling effect that restricted further incremental symmetry gains from nGVS, which logically supported our finding that individuals with greater spinal deformities derived more pronounced biomechanical benefits. Similar ceiling phenomenon has been observed in healthy young adults, where nGVS yielded no significant enhancement in balance control during standing tasks when baseline vestibular function was intact (Assländer et al., 2021). Furthermore, the specific symmetry metrics correlated with Cobb angle varied between stair ascent and descent, reflecting the distinct biomechanical demands of each task. Taken together, nGVS did not exert uniform effects on gait symmetry across varying spinal deformity severities in AIS; rather, its biomechanical efficacy was state-dependent, scaling with the severity of scoliosis within the mild-to-moderate range.

### Limitations

Several limitations of this study warrant consideration. First, VPT was employed exclusively to calibrate individualized nGVS intensity and was not utilized to assess vestibular function, thereby restricting investigating the relationship between nGVS efficacy and the baseline severity of vestibular dysfunction. Future studies should incorporate comprehensive vestibular assessments (*e.g.*, video head impulse test and vestibular evoked myogenic potentials) to address this gap. Second, the repetitive nature of the experimental protocol may have introduced learning or fatigue effects that could interact with nGVS-induced neuromodulation, despite the randomized trial sequence (Wrisley et al., 2007). Third, the present study did not include a healthy control group. Consequently, although nGVS significantly improved medial-lateral gait symmetry during stair negotiation in AIS, it cannot be definitively determined whether these improvements represent a true biomechanical normalization toward able-bodied gait patterns or merely an alteration of symmetry metrics without clinical significance. Future clinical trials incorporating healthy controls are warranted to establish normative baseline benchmarks and confirm the therapeutic value of nGVS-induced gait symmetry modulation. Fourth, walking speed and step width, established modulators of gait symmetry, were self-selected rather than systematically controlled. Although participants were instructed to maintain a consistent, comfortable gait pattern across conditions, uncontrolled inter-condition variations in these parameters may have influenced gait performance. Future work should statistically control for these covariates to isolate the specific neuromodulatory effects of nGVS. Fifth, a between-trial protocol alternating the leading foot (convex-side *vs.* concave-side) was employed to construct symmetry metrics, which may introduce subtle variations in stepping strategy across sides. Multi-step stair protocols allowing for within-trial comparisons is warranted to address this limitation. Sixth, specific data processing and analytical approaches constrained certain biomechanical and statistical interpretations. Although applying unified cut-off frequencies prevented spurious waveform fluctuations and aligned with established gait analysis standards, filtering inherently altered high-frequency signal spectra (Sinclair, Taylor & Hobbs, 2013). Similarly, evaluating Xcorr at the optimal time lag isolated spatial waveform similarity, but reduced sensitivity to temporal phase asymmetries (Wren et al., 2006). Furthermore, while restricting Cobb-angle correction analyses to pre-screened variables effectively controlled multiplicity, this strategy carried inherent risks of selection bias, regression-to-the-mean, and potential overestimation of correlation strength (Kriegeskorte et al., 2009). Finally, although frontal-plane instability represents the primary driver of medial-lateral gait symmetry in AIS, scoliosis is intrinsically a three-dimensional deformity. Future studies should incorporate multi-planar symmetry analyses, particularly horizontal (yaw-plane) axial rotation, to further elucidate whether nGVS demonstrates distinct neuromodulatory benefits across different planes.

## Conclusions

In summary, nGVS significantly improved medial-lateral gait symmetry in patients with AIS during stair negotiation, but not during level walking. These task-specific symmetry improvements, observed across both kinematic and kinetic domains, suggested a potential refinement of individual segmental control across the spine-pelvis-hip complex and global kinetic dynamics during tasks under heightened biomechanical demands. Furthermore, nGVS-induced symmetry enhancements demonstrated a state-dependent pattern within the mild-to-moderate range (Cobb angle 10°–30°), wherein patients exhibiting larger Cobb angles derived more pronounced symmetry gains. Whether these findings extend to severe spinal deformities (>30°) warrants further investigation.

## Supplemental Information

10.7717/peerj.21947/supp-1 Table S1 Summary of symmetry indices (SI) and normalized cross-correlation coefficients (Xcorr) for kinematic and kinetic parameters Data were presented as mean ± standard deviation. ML1, first ground reaction force (GRF) peak in the medial-lateral (ML) direction; ML2, second GRF peak in the ML direction; nGVS, noisy galvanic vestibular stimulation; No, no stimulation; P, lateral pelvic displacement; Sham, sham stimulation; T, lateral trunk displacement; *α*, lateral trunk lean; *β*, pelvic obliquity; *θ*, hip adduction.

10.7717/peerj.21947/supp-2 Table S2 Summary of peak values of kinematic and kinetic parameters from convex-side and concave-side limbs across different conditions Data were presented as mean ± standard deviation. ML1, first ground reaction force (GRF) peak in the medial-lateral (ML) direction; ML2, second GRF peak in the ML direction; nGVS, noisy galvanic vestibular stimulation; No, no stimulation; P, lateral pelvic displacement; Sham, sham stimulation; T, lateral trunk displacement; *α*: lateral trunk lean; *β*, pelvic obliquity; *θ*, hip adduction.

10.7717/peerj.21947/supp-3 Table S3 Summary of symmetry indices (SI) for kinematic parameters without static offset correction and respective statistical results from the two-way repeated-measures ANOVA for sensitivity analysis Data were presented as mean ± standard deviation. *df*, degrees of freedom; nGVS, noisy galvanic vestibular stimulation; No, no stimulation; P, lateral pelvic displacement; Sham, sham stimulation; T, lateral trunk displacement; *α*, lateral trunk lean; *β*, pelvic obliquity; *θ*, hip adduction. **, *p* < 0.01.

10.7717/peerj.21947/supp-4 Data S1 Datasets for gait kinematics/kinetics and respective symmetry data The raw data file showed all processed gait kinematics/kinetics and respective symmetry data for statistical analysis.

10.7717/peerj.21947/supp-5 Supplemental Information 5 CONSORT Checklist

10.7717/peerj.21947/supp-6 Supplemental Information 6 Trial protocol—English

10.7717/peerj.21947/supp-7 Supplemental Information 7 Trial protocol—Original Chinese

## Additional Information and Declarations

### Data Availability

The following information was supplied regarding data availability: The raw data are available in the Supplemental Files.
