# Supplementary material for: Task-specific effects of noisy galvanic vestibular stimulation on gait symmetry in adolescent idiopathic scoliosis
Source: PeerJ. 2026 Nov 12;14:e21947. doi: 10.7717/peerj.21947 (PMC13770426; doi:10.7717/peerj.21947)
Supplement: Supplementary file 2 [file peerj-14-21947-s006.pdf]

Clinical Trial Protocol

Project Title: Biomechanical Effects and Neuromodulation Mechanisms of Proprioceptive Combined with Vestibular Stimulation on Gait Performance and Postural Control in Adolescents with Idiopathic Scoliosis

Version No.: V 2.0

Date: January 25th, 2026

2. Study Objectives

Primary Objective:

To investigate the immediate synergistic effects of combined proprioceptive and vestibular stimulation on postural control and gait biomechanics in patients with adolescent idiopathic scoliosis (AIS). By quantitatively comparing key biomechanical metrics—including postural sway parameters, gait symmetry, joint coordination, and dynamic margin of stability—across four intervention groups, this study aims to reveal sensory integration deficits in patients with AIS and to elucidate the independent and synergistic roles of these two sensory stimulation modalities in enhancing posture and gait.

Secondary Objective:

To uncover the central and peripheral neural integration mechanisms by which combined stimulation regulates posture and gait in patients with AIS. Specifically, this study aims to clarify how combined stimulation modulates the integrative function of the sensorimotor cortex and the vestibulospinal pathway to optimize muscle control strategies, thereby delineating the central nervous system mechanisms underlying improved postural control and gait performance.

3. Study Design

This is an interventional, randomized controlled trial. Eligible participants will be randomly assigned to four parallel groups in a 1:1:1:1 ratio, receiving clinical interventions 3 times per week for 8 weeks, followed by a 6-month longitudinal follow-up. Participants will be recruited from the Department of Rehabilitation Medicine at Suzhou Municipal Hospital through poster advertisements and clinical referrals.

Random sampling and allocation will be performed using an envelope method for

potential participants meeting the inclusion and exclusion criteria. A block randomization list (block size of 4 or 8) will be generated using SPSS software by an independent researcher not involved in intervention delivery, outcome assessment, or participant recruitment. Allocation concealment will be secured using sequentially numbered, sealed, opaque envelopes. Upon obtaining written informed consent and completing baseline evaluations, the investigator will open the corresponding envelope sequentially to determine group assignment.

(1) Intervention Groups

- **Experimental Group A (Vestibular Stimulation / nGVS Group)**: Participants will receive noisy galvanic vestibular stimulation (nGVS) applied to the bilateral vestibular system using a portable current stimulator (BC102-IV, BrainCLOS Ltd., China). A pair of circular silicone electrodes with a surface area of 1.75 cm^2 will be placed over the bilateral mastoid processes and secured with medical breathable tape. Compared with conventional devices, these smaller electrodes offer more precise stimulation targeting and higher current density. The nGVS delivery parameters comprise a frequency bandwidth of 4-20 Hz, an intensity set to 80% of the individual's vestibular perceptual threshold (VPT) (subthreshold stimulation), and a session duration of 40 minutes. Because an intensity of 80% VPT is subthreshold, participants will not experience subjective perception of electrical stimulation. Preliminary studies have confirmed that these parameters effectively induce stochastic resonance, activating primary otolith and semicircular canal neurons within the vestibular system to modulate vestibular afferent signaling.

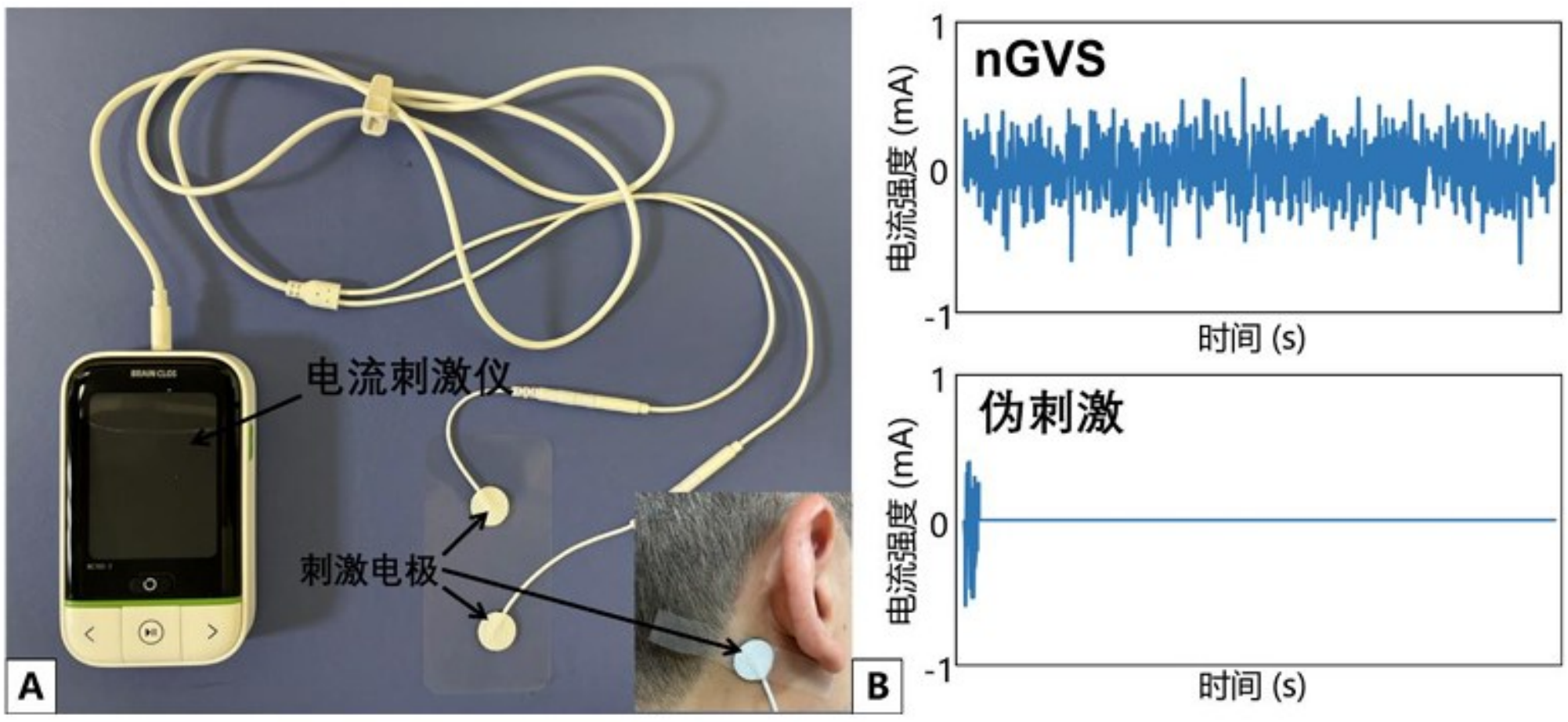

- **Experimental Group B (Proprioceptive Stimulation Group)**: Participants will receive subthreshold plantar proprioceptive stimulation via C-2 tactor actuators (Engineering Acoustics Inc., Florida, USA) embedded in custom insoles. Vibration parameters include a frequency of 250 Hz, an intensity set to 130% of the individual's vibration perceptual threshold, and a duty cycle of 0.9 seconds (0.3s

vibration period followed by a 0.6s pause). Interventions will last 40 minutes per session.

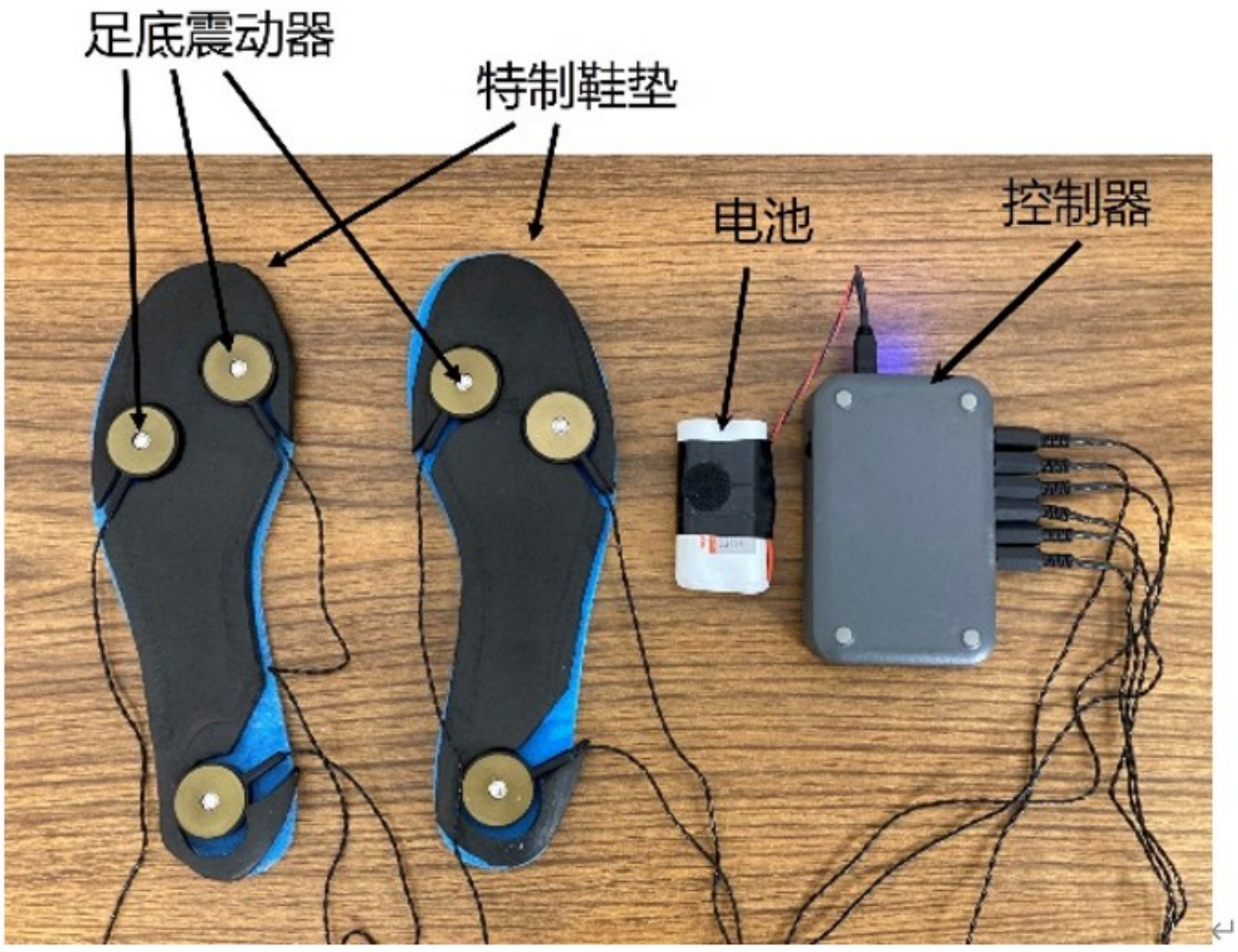

- **Experimental Group C (Combined Proprioceptive and Vestibular Stimulation Group)**: Participants will receive synchronous nGVS and plantar vibration stimulation using the aforementioned devices and settings for 40 minutes per session.
- **Experimental Group D (Control Group)**: Participants will be fitted with identical devices (nGVS and plantar vibration insoles) placed in the same locations with identical parameters. However, the current or vibration output will be turned off (0 mA or 0 dB) after 30 seconds of initial ramp-up to simulate active stimulation while maintaining sham conditions. Interventions will last 40 minutes per session.

4. Participant Selection

4.1 Inclusion Criteria

- Radiographically confirmed coronal spinal curvature with a Cobb angle between 10° and 25°.
- Age between 10 and 17 years.
- Risser sign grade 0–3.
- Positive Adam's forward bend test.

4.2 Exclusion Criteria

- Presence of spinal deformities of known secondary etiology (e.g., congenital, neuromuscular).
- History of low back pain within the preceding 3 months.
- History of spinal trauma or spinal surgical interventions.
- Traumatic injury to the trunk or lower limbs within the past 6 months.
- Co-occurring neuromuscular, vestibular, or orthopedic conditions affecting gait, movement, or balance.
- Abnormal or absent plantar cutaneous sensation.
- Inability to ambulate independently without assistive devices (e.g., canes, walkers).

4.3 Discontinuation Criteria

- Withdrawal of informed consent by the participant or their legal guardian.
- Decision by the principal investigator to withdraw the participant for safety or compliance reasons in their best interest (e.g., clinical disease progression, persistent non-compliance).

4.4 Sample Size Calculation

Sample size was calculated using G*Power software. Based on 4 parallel study groups, an analysis of variance (ANOVA) effect size (f=0.45) was determined using a Partial Eta Squared (η^2_p =0.157). A minimum sample size of 17 participants per group is required to achieve 80% statistical power (α=0.05). Accounting for an estimated 15% drop-out rate, 20 participants will be enrolled per group, yielding a total sample size of 80 participants (N=80).

5. Primary Outcome Measures

Assessments will be conducted at baseline (pre-intervention) and post-intervention, capturing clinical manifestations, vestibular function, paraspinal muscle structural/functional parameters, functional near-infrared spectroscopy (fNIRS) signals, and surface electromyography (sEMG) data. Follow-up evaluations at Month 3 and Month 6 will re-assess clinical manifestations and paraspinal muscle status.

5.1 Clinical Manifestations

- Scoliotic Angle (Cobb Angle): Measured on standard full-length standing anteroposterior spinal radiographs obtained within 1 week of each evaluation

timepoint. A specialized physician will determine the Cobb angle using UniWeb Viewer 8.0 software (EBM Technologies, China) by identifying the upper and lower end vertebrae (most tilted toward the concavity), drawing parallel lines along the superior endplate of the upper end vertebra and inferior endplate of the lower end vertebra, and measuring the angle formed by their perpendicular intersections.

- **Clinical Scales:**
  1. Scoliosis Research Society-22 Questionnaire (SRS-22): The simplified Chinese version of the SRS-22 will evaluate health-related quality of life across 5 domains (Function, Pain, Self-Image, Mental Health, and Treatment Satisfaction) using 22 items rated on a 5-point Likert scale (1 = lowest, 5 = highest). Higher total scores indicate superior quality of life.
  2. Spinal Appearance Questionnaire (SAQ): The simplified Chinese version of the SAQ will assess self-perceived spinal appearance across 8 domains and 16 items (scored 1 to 5; maximum total score = 80). Higher scores denote greater dissatisfaction with spinal appearance.

5.2 Vestibular Function

- Cervical Vestibular Evoked Myogenic Potentials (c-VEMP): Evaluated using an electromyographic/evoked potential system (MEB-9404c, NIHON KOHDEN Corp., Japan) with galvanic stimulation to test the integrity of the sacculo-collic / vestibulocollic reflex pathway.
- Vestibular Perceptual Threshold (VPT): Defined as the minimum current intensity evoking subjective perception of head movement or postural shift. Galvanic vestibular stimulation delivered via a BC102-IV stimulator (BrainCLOS Ltd., China) will be coupled with standing center-of-gravity (CoG) sway tracking using an AMTI-HPS 3D force plate (Advanced Mechanical Technology Inc., USA) to determine individual VPT values.

5.3 Paraspinal Muscle Structure and Function

- Surface Electromyography (sEMG): Paraspinal muscle activation asymmetry (convex vs. concave sides) during motor tasks will be captured at 2000" Hz" using a wireless sEMG system (Delsys Inc., USA) synchronized with kinetic/kinematic hardware via a DELSYS Trigger Module.
- Musculoskeletal Ultrasound: B-mode imaging using a portable ultrasound system (SONIMAGE HS1, KONICA MINOLTA Inc., Japan) will measure resting and maximum contraction muscle thickness of bilateral paraspinal muscles at the upper

end vertebra, apex, and lower end vertebra. Images will be quantified using ImageJ software.

5.4 Static Postural Control

Kinetic data collected via a 3D force plate during quiet standing tasks will yield center of pressure (COP) parameters:

- COP Trajectory Length: Total sway path length (mm) during quiet standing.
- COP Velocity: Mean sway velocity in anterior-posterior (AP) and medial-lateral (ML) directions (mm/s).
- 95% Confidence Ellipse Area: Surface area of the ellipse enclosing 95% of the COP trajectory (mm^2), alongside AP and ML major axis lengths (mm).
- COP Variability: Quantified using the Coefficient of Variation (CV=Standard Deviation/Mean) and Sample Entropy (SaEn) across COP variables.

5.5 Dynamic Postural Control

Kinematic data during locomotion will be recorded using an 8-camera Vicon 3D motion capture system (VICON Inc., Oxford, UK) synchronized with Qualisys Tracker Manager software. Walking tasks will be performed on a multi-incline bidirectional treadmill (Advanced Mechanical Technology Inc., USA) under two paradigms: flat walking (0% grade) and inclined walking (+8%/+15% incline, -8%/-15% decline). Participants will walk at their preferred walking speed (PWS) for 2 minutes per task, followed by 2 minutes of rest. Evaluated parameters include:

- Spatiotemporal Parameters: Step length (m), step time (s), stride length (m), stride time (s), and step width (m).
- Gait Stability: Margin of Stability (MOS) calculated in AP and ML directions (m).
- Joint Kinematics: Bilateral hip, knee, and ankle joint range of motion (degree) and peak joint angles (degree) throughout the gait cycle.
- Gait Variability: Quantified via Coefficient of Variation (CV) and largest Lyapunov Exponent (λ). Values approaching 0 (CV) or $\lambda \leq 0$ indicate lower variability and superior dynamic stability.

6. Secondary Outcome Measures

6.1 Functional Near-Infrared Spectroscopy (fNIRS)

Cortical hemodynamic responses, specifically oxygenated hemoglobin (HbO) concentrations, will be continuously recorded during motor tasks using a 65-channel fNIRS cap system (Yirui Inc., Wuhan, China) operating at 730–850 nm wavelengths with an 11 Hz sampling frequency. Optodes will be positioned according to the international 10-20 EEG system with probe D3 anchored at Fpz and an optode-detector distance of 3 cm, covering frontal, parietal, and occipital regions. Regions of Interest (ROIs) include bilateral:

- Dorsolateral Prefrontal Cortex (DLPFC, Brodmann Area 9/46)
- Supplementary Motor Area (SMA, Brodmann Area 6)
- Primary Motor Cortex (M1, Brodmann Area 4)
- Primary Somatosensory Cortex (S1, Brodmann Area 1/2/3)
- Vestibular Cortex (VC, Brodmann Area 2v/7)

fNIRS Paradigms:

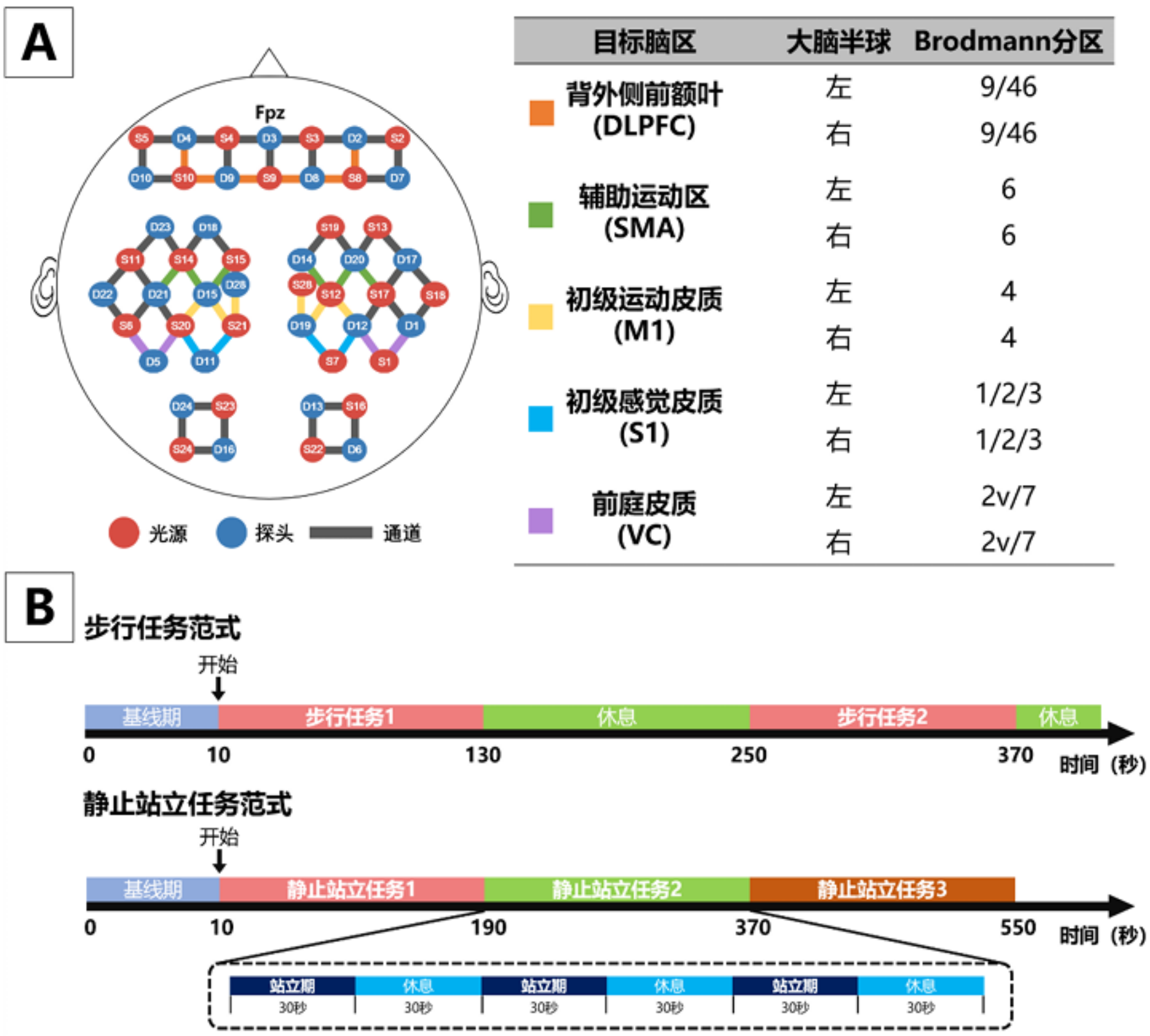

7. Follow-up Plan

Longitudinal follow-up evaluations will be performed at Week 12 (3 months) and Week 24 (6 months) following intervention completion to assess the sustainability of treatment effects on clinical scales and paraspinal muscle characteristics.

8. Statistical Analysis

Primary metrics include Cobb angle, clinical scale scores, vestibular parameters, paraspinal muscle parameters, cortical activation levels, and functional connectivity metrics across sensorimotor ROIs.

- Intervention Comparisons: Repeated-measures Analysis of Variance (rmANOVA) will be performed to evaluate pre- to post-intervention changes, treating evaluation timepoints as the within-subject factor and intervention groups (Groups A–D) as the between-subject factor.
- Correlation Analyses: Bivariate association analyses will use Pearson correlation coefficients for continuous, normally distributed variables, and Spearman rank correlation coefficients for ordinal or non-normally distributed data.

9. Adverse Event Handling

This trial utilizes non-invasive neuroimaging (fNIRS), non-invasive neuromodulation (nGVS), and peripheral sensory stimulation (plantar vibration). The incidence of adverse events with these modalities is extremely low (no adverse events reported in preliminary studies). Should any unexpected adverse event occur, it will be handled immediately and reported to the Institutional Review Board (IRB) in strict compliance with ethical regulations. Personal and psychological safety risks during data collection will be minimized through strict data encryption, anonymization, and confidentiality protocols.

10. Data Collection and Management

Data will be captured electronically using dedicated software platforms:

- fNIRS Data: NIRStar workstation software
- sEMG Data: BTS FreeEMG 300 software suite
- Kinematic & Kinetic Data: Qualisys Tracker Manager software

All raw metrics will be collated, archived, and managed in secure spreadsheets. Custom MATLAB scripts will be executed to synchronize and integrate multi-modal data streams (fNIRS, sEMG, kinematics, and kinetics). All datasets will be stored on dedicated, password-protected laboratory workstations with restricted access privileges.
