# Supplementary material for: Task-specific effects of noisy galvanic vestibular stimulation on gait symmetry in adolescent idiopathic scoliosis
Source: PeerJ. 2026 Nov 12;14:e21947. doi: 10.7717/peerj.21947 (PMC13770426; doi:10.7717/peerj.21947)
Supplement: Supplementary file 3 [file peerj-14-21947-s007.pdf]

版本号：V 2.0

版本定稿时间：2026 年 1 月 25 日

临床试验方案

1. 研究项目名称：本体感觉联合前庭刺激对青少年特发性脊柱侧弯患者步态表现及姿势控制的生物力学影响及神经调控机制研究

2. 研究目的：

主要目标：探究本体感觉与前庭联合刺激对青少年特发性脊柱侧凸（AIS）患者姿势控制与步态生物力学的即时协同效应。定量比较 4 组受试者的姿势摇摆参数、步态对称性、关节协调性、动态稳定性边际等关键生物力学指标，揭示 AIS 患者的感觉整合缺陷，并明确两种感觉刺激在改善姿势与步态中的独立与协同作用。

次要目标：揭示联合刺激调控 AIS 患者姿势与步态的中枢-外周神经整合机制。阐明联合刺激如何通过调节感觉运动皮层的整合功能与前庭-脊髓通路，最终优化肌肉控制策略，从而改善 AIS 患者姿势与步态的中枢神经机制。

3. 研究设计：

本研究为一项干预性研究，受试者按照 1：1：1：1 的比例随机分配到 4 组，并接受 3 次/周，为期 8 周的临床干预和 6 个月的纵向随访。计划从苏州市立医院康复医学科就诊的 AIS 患者中招募受试者。

本研究采用信封法的方式对符合纳入/排除标准的潜在受试者进行随机抽样（入组 vs 不入组）。受试者按照 1∶1∶1∶1 的比例随机分配至四个组别。随机分配序列由一名不参与实验干预、受试者评估及招募工作的专业实验人员通过 SPSS 软件生成区组长度为 4 或 8 的区组随机化列表。随机分组信息采用按序编号、密封、不透光的信封进行分配隐藏。研究者在获取受试者知情同意并完成基线评估后，按入组顺序依次开启对应信封，确定分组。

实验组 A（前庭刺激组）：采用便携式电流刺激仪（BC102-IV, BrainCLOS Ltd., China）对 AIS 受试者的双侧前庭系统施加随机噪声电刺激（noisy galvanic vestibular stimulation, nGVS）。将一对表面积为 1.75 cm^2 的圆形硅胶电极放置于 AIS 患者双侧耳后乳突处，并使用医用透气胶带进行固定。该设备的电极较传统仪器更小，能够实现更精准的刺激定位，并提供更高的电流密度。本研究中，nGVS 的电流频率范围为 4-20 Hz，电流强度为 AIS 受试者个体前庭感知阈（vestibular perceptual threshold, VPT）的 80%，干预时长为 40 分钟。由于强度为 80% VPT 的 nGVS 属于阈下刺激，受试者在干预过程中不会主观感知到电刺激的施加。申请人的前期研究已证实，本实验采用的 nGVS 电流参数能够有效诱发随机共振效应，激活前庭系统的初级耳石和半规管神经元，从而调控前庭传入信号。

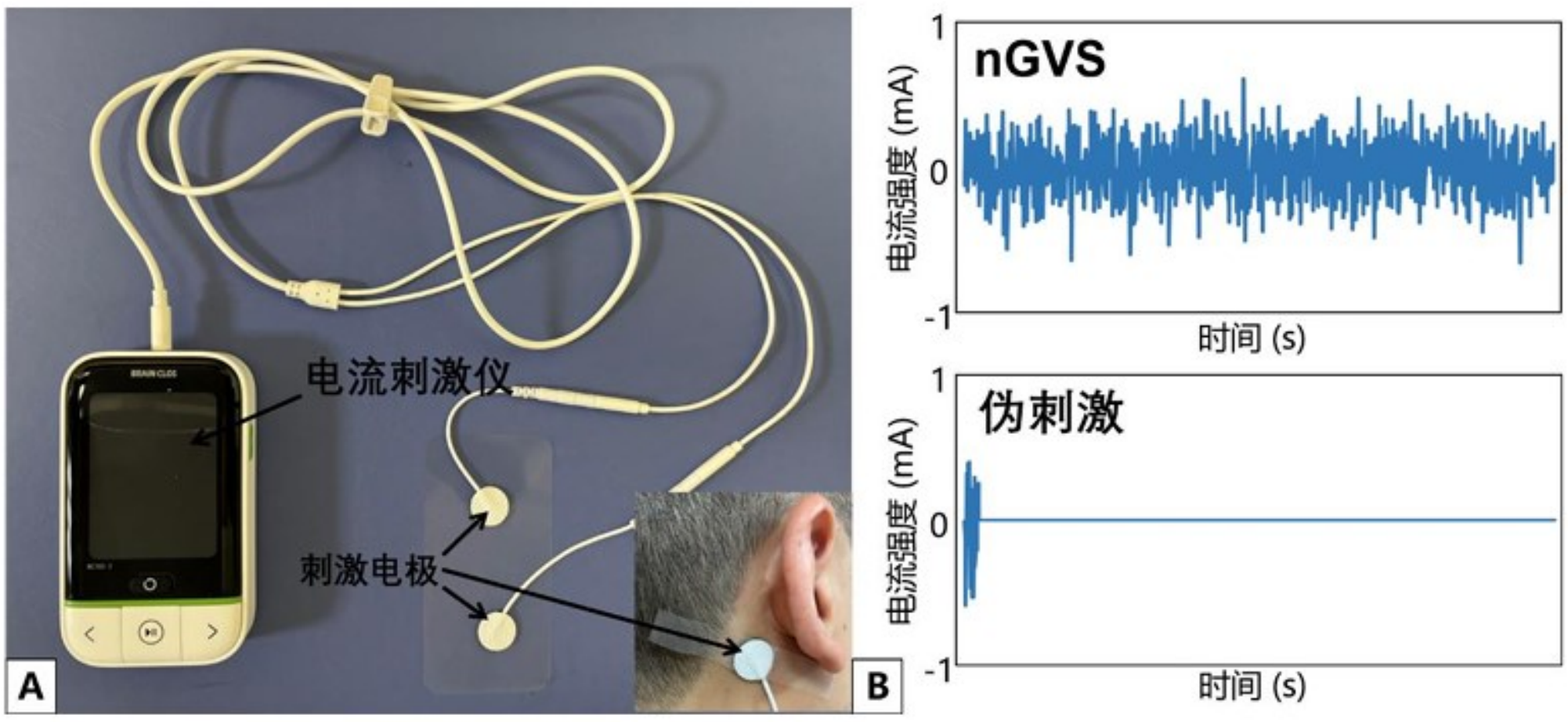

实验组 B（本体感觉刺激组）： 采用 C-2 震动传感器（Engineering Acoustics Inc.，佛罗里达，美国）对 AIS 患者的足底区域提供本体感觉刺激。震动传感器固定于特制鞋垫中，震动频率为 250Hz，震动强度为受试者个体振动觉阈值（vibration perceptual threshold）的 130%，震动周期为 0.9 秒，其中 0.3 秒为震动期、0.6 秒为间歇期。干预时长为 40 分钟。

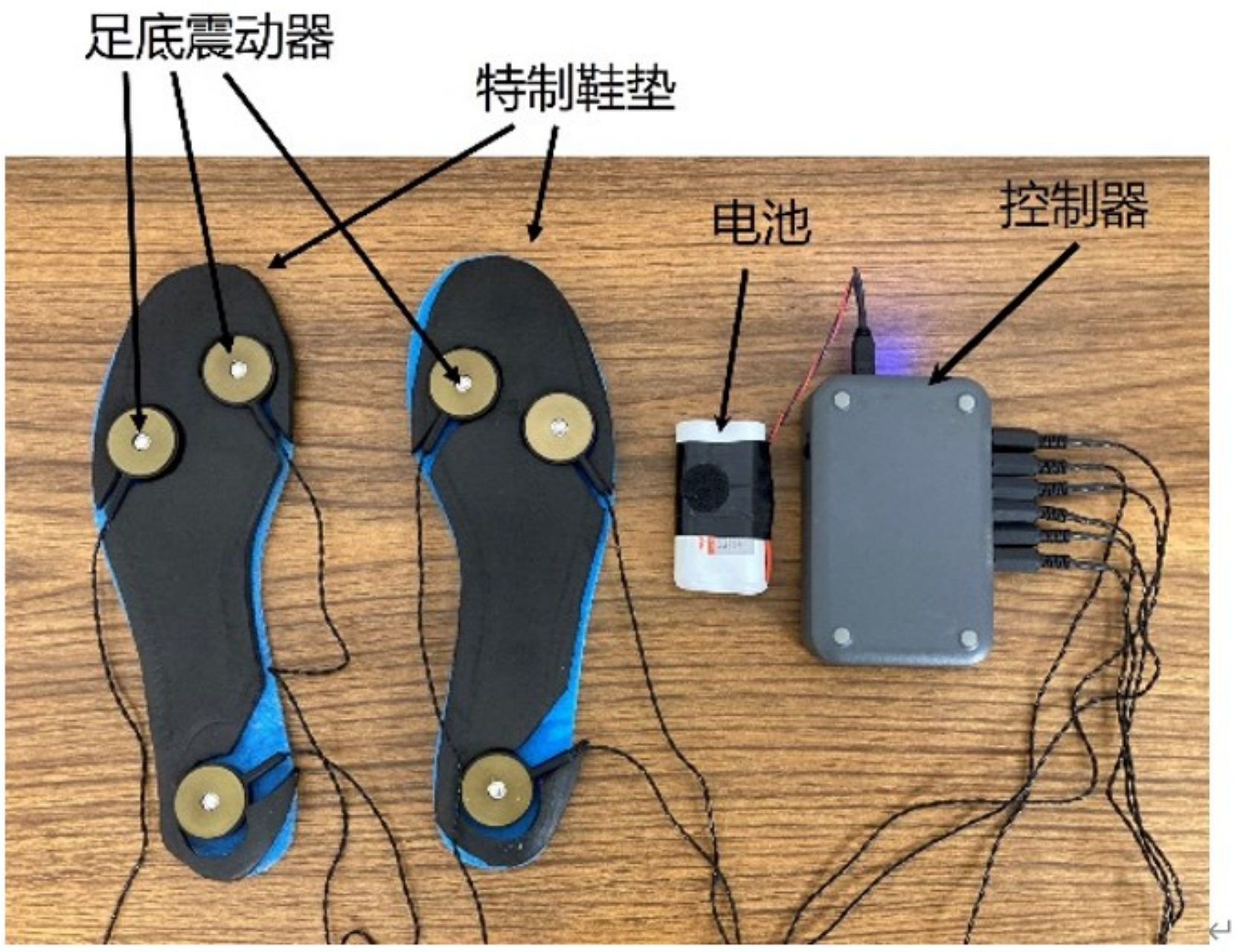

实验组 C（本体感觉联合前庭刺激组）： 使用上述 nGVS 设备与足底震动设备同步对 AIS 患者进行本体感觉联合前庭刺激。干预时长为 40 分钟。

实验组 D（对照组）： 伪刺激所采用的干预设备（nGVS、足底震动）、放置方法及干预参数与上述实验组相同，但在设备开机 30 秒后关闭电源（强度为 0 mA 或 0 dB）。干预时长为 40 分钟。

4. 病例的选择：

4.1 纳入标准：

1）通过 X 光影像检查，明确存在脊柱冠状面侧向弯曲且 Cobb 角为 10°-25°

之间；2）年龄在 10-17 周岁；3）Risser 征为 0-3 级；4）Adam 躯干前屈试验为阳性。

4.2 排除标准：

1）存在任何已知原因的脊柱畸形；2）过去三个月内存在下背痛；3）存在脊柱外伤史或手术史；4）过去六个月内存在躯干或下肢的创伤性损伤； 5）存在任何影响步态、运动、姿势的疾病；6）存在足底皮肤感觉异常或缺失；7）在无辅助器具（如手杖、助行器）的情况下无法独立完成步行。

4.3 终止标准：

1）受试者撤回知情同意书。2）研究者认为出于安全性和/或依从性的原因，出于受试者的最大利益考虑让其退出，如病情恶化、患者不配合试验等。

4.4 样本量计算：

本实验的样本量通过 G*Power 统计软件计算得出。根据本实验的组别设定（4 组），使用 Partial Eta Squared 方法确定方差分析效应量（η^2=0.157）和统计效应（effect size f=0.45）。计算后得到每组样本量需达到 17 人以实现 80%的统计检验力，另参考 15%的脱落率，每组需纳入 20 例受试者，因此本实验的总样本量为 80 例。

5. 观察指标：

本研究将在干预前、干预后对每位 AIS 受试者的临床表现、前庭功能、椎旁肌结构/功能以及 fNIRS 信号数据、EMG 信号数据进行评估。此外，在随访的第 3 个月和第 6 个月时，将重复进行临床表现和椎旁肌结构/功能评估。本研究的具体评估方法如下所述：

5.1 临床表现评估

本研究的临床表现评估包含脊柱形态与临床量表两部分。具体介绍如下：

1）脊柱侧凸角度

本研究通过拍摄脊柱标准全长正位 X 线片测量 Cobb 角，以评估 AIS 患者脊柱侧凸的严重程度。脊柱标准全长正位 X 线片是诊断和评估 AIS 的“金标准”。为确保评估的准确性，本研究要求 AIS 患者在每个评估时间点前后一周内完成 X 线片的拍摄。Cobb 角的测量由一名专业医师使用 UniWeb Viewer 8.0（EBM Technologies, China）软件完成，具体步骤如下：1. 确定上、下端椎，即向脊柱侧弯凹侧倾斜度最大的椎体；2. 分别在上端椎的上缘和下端椎的下缘划平行线，并作垂线；3. 两垂线的交角即为 Cobb 角。

2）临床量表评估

a. 脊柱侧凸研究学会 22 项量表（Scoliosis Research Society-22, SRS-22）：本

研究采用简体中文版 SRS-22 对 AIS 患者的健康状况和生活质量进行评估。SRS-22 量表包括 22 个题目，分为 5 个维度（功能、疼痛、自我形象、心理健康和治疗满意度）。SRS-22 量表采用 Likert 5 级评分法，每个问题的得分范围为 1 分（最低）至 5 分（最高），所有题目得分的总和即为量表总分。总分越高，代表 AIS 患者的健康状况和生活质量越好。既往研究已证实，SRS-22 量表具有良好的信度和效度（Cronbach's α=0.79~0.85，ICC=0.77~0.87），能够有效评估 AIS 患者的健康状况与生活质量。

b. 脊柱外观问卷（spinal appearance questionnaire, SAQ）：本研究采用简体中文版 SAQ 对 AIS 患者的脊柱外观进行评估。SAQ 共包含 8 个维度和 16 个计分项目，每个项目的分值范围为 1 分（最低）至 5 分（最高），总分为 80 分。分值越高，表明患者对自我外观的满意度越低。既往研究证实，中文版 SAQ 具有良好的信度与效度（Cronbach's α=0.521~0.792，ICC=0.965），能够全面评估 AIS 患者的外观表现。

5.2 前庭功能评估

本研究的前庭功能评估包含 c-VEMP 和 VPT 检测两部分。具体介绍如下：

1）颈肌前庭诱发肌源性电位（c-VEMP）

前庭诱发肌源性电位是一种客观、无创的神经电生理检测技术，广泛应用于前庭系统通路完整性的检测及前庭系统疾病的诊断。其中，c-VEMP 能够反映前庭-颈反射通路（vestibulocollic reflex）的完整性，该通路起源于球囊，通过前庭下神经传递至脑干的前庭神经核，再经由内侧纵束下行至同侧副神经核，最终诱发同侧胸锁乳突肌的抑制性电位。本研究采用肌电诱发电位仪（MEB-9404c, NIHON KOHDEN Corp., Japan）施加乳突直流电刺激以诱发 c-VEMP，从而评估 AIS 患者的前庭功能。

2）前庭感知阈（VPT）

VPT 是指能够引起个体对头部运动或位置变化感知的最小刺激强度，反映了前庭系统对运动或外界刺激的敏感程度。VPT 是评估前庭系统感知功能的重要指标，在衰老及前庭相关疾病的诊断中具有重要作用。本研究采用便携式电流刺激仪（BC102-IV, BrainCLOS Ltd., China）施加前庭电刺激，并借助 AMTI-HPS 型三维测力台（Advanced Mechanical Technology Inc., USA）采集站立位的重心（CoG）位移数据，从而评估 AIS 患者的 VPT。

5.3 椎旁肌结构与功能评估

本研究的椎旁肌结构与功能评估包含 sEMG 和肌骨超声两部分。具体介绍

如下：

1）椎旁肌表面肌电（sEMG）信号

本研究通过评估 AIS 患者执行运动任务过程中脊柱两侧椎旁肌的 sEMG 信号，以分析 AIS 患者凸/凹侧椎旁肌的失衡状态。本研究使用便携式无线 sEMG 系统（Delsys Inc., USA）采集 AIS 患者脊柱两侧椎旁肌的 sEMG 信号，采样频率设置为 2000 Hz。sEMG 系统通过 DELSYS Trigger Module 与三维运动分析系统（测力台、动作捕捉系统）相连，实现 sEMG 信号与动力学/运动学数据的同步采集。

2）肌骨超声

本研究采用便携式康复超声诊断系统（SONIMAGE HS1, KONICA MINOLTA Inc., Japan）评估 AIS 受试者脊柱两侧椎旁肌的形态结构。该设备支持 双窗口同屏显示功能。使用亮度模式（B-mode）分别在静息和最大收缩状态下， 对 AIS 患者脊柱侧凸上端椎体、下端椎体和顶椎两侧的椎旁肌进行图像采集。使用 Image J 图像分析软件对超声图像进行处理，并测量两侧椎旁肌的静息厚度和收缩厚度。

5.4 静态姿势控制评估

本研究通过三维测力台（force plate）采集 AIS 患者执行静止站立任务过程中的动力学数据，分别评估其静态姿势控制能力。通过以下 COP 参数评估 AIS 受试者在静止站立任务中的姿势控制表现：

1）COP 位移轨迹长度：指静止站立任务过程中 COP 位移所形成运动轨迹的总长度（mm）。

2）COP 位移速度：指静止站立任务过程中 COP 在前-后方向及内-外方向上的位移速度（mm/s）。

3）95%置信椭圆：指静止站立任务过程中 COP 运动轨迹的 95%置信区间所围成的椭圆面积（mm2），以及椭圆前-后轴和内-外轴的长度（mm）。

4）COP 位移变异性：通过变异系数（coefficient of variance, CV=均值/标准差）和样本熵值（sample entropy, SaEn）评估上述 COP 参数在静止站立 任务中的变异性。

5.5 动态姿势控制评估

本实验拟使用 Vicon 三维运动捕捉系统（VICON Inc.，牛津，英国）获取步

态与姿势控制相关的运动学数据。该系统包含 6 个红外高清摄像头，可从多角度采集反光标记物的空间位置坐标及运动轨迹。坐标数据将在配套软件 Qualisys Tracker Manager 中模拟三维的人体步行运动轨迹。本研究的步行任务通过多坡度双向跑步机（Advanced Mechanical Technology Inc., USA）实现，共包括平地步行（0%）和斜坡步行（8%/15%上坡、-8%/-15% 下坡）两种运动范式。在每次步行任务中，AIS 受试者以最适步行速度 （preferred walking speed, PWS）在跑步机上进行 2 分钟步行，随后休息 2 分钟以恢复至静息状态。本研究通过以下步态参数评估 AIS 受试者的步态表现：

1）时-空间步态特征：包括步长（step length, m）、步时（step time, s）、 跨步长（stride length, m）、跨步时间（stride time, s）和步宽（step width, m）。

2）步态稳定性：使用稳定边际（margin of stability, MOS）对步态稳定性进行评价，包括前-后方向和内-外方向的 MOS（m）。

3）下肢关节角度：包括步态周期中双侧髋关节、膝关节和踝关节的活动度（°）及其峰值（°）。

4）步态变异性：使用变异系数（CV）和 Lyapunov 指数（λ）计算上述步态参数的变异性。当 CV 趋近于 0 或 λ≤0，表明步态变异性较小，受试者的步态表现较好。

5.6 功能性近红外脑成像（fNIRS）

在执行运动任务的过程中，本研究采集 65 通道 fNIRS 设备（依瑞德，武汉，中国）同步采集 AIS 受试者的血流动力学数据（含氧血红蛋白（oxygenated hemoglobin, HbO）浓度）。fNIRS 数据采集模块的光源和探头放置参考国际脑电图 10-20 电极放置标准，标准点设定为 D3 探头（Fpz 点），光源与探头间距为 3 cm，覆盖额叶、顶叶及部分枕叶。 数据采集通过 NIRScan 软件程序进行，fNIRS 设备的采集波长设定为 730-850 nm，采样频率为 11 Hz。本研究的主要观察指标为 HbO 浓度（该指标对局部脑血氧变化最为敏感）；其值越高，表明该脑区含氧量越丰富，间接提示脑部激活程度较高。本研究的感兴趣区域（regions of interest, ROIs）包括双侧大脑半球的背外侧前额叶（dorsolateral prefrontal cortex, DLPFC）、初级感觉皮层（primary sensory cortex, S1）、初级运动皮层（primary motor cortex, M1）、辅助运动区（supplementary motor area, SMA）和前庭皮质（vestibular cortex, VC）。

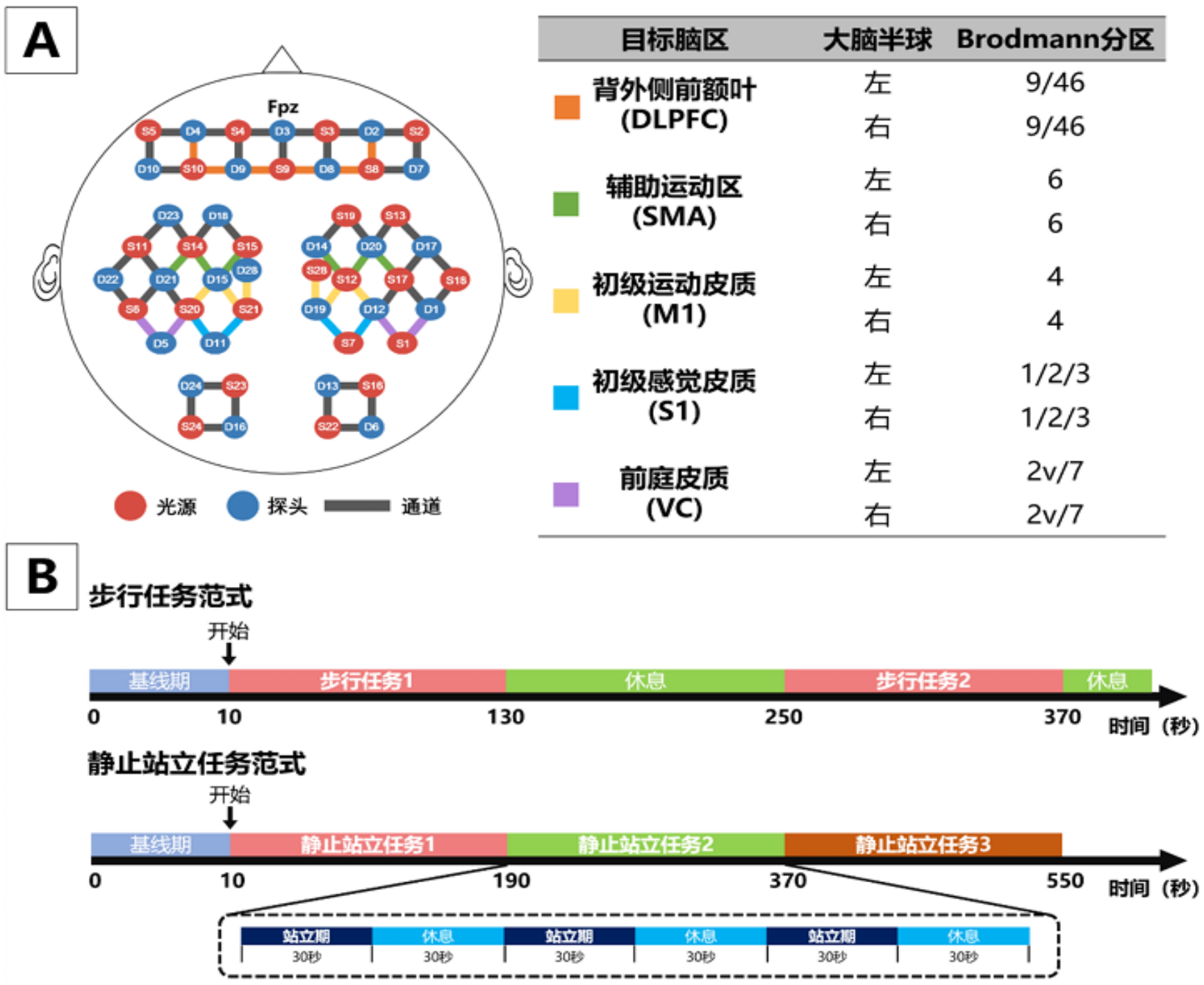

本实验所采用 fNIRS 评估的运动范式包含两个部分。针对平地和斜坡步行任务，运动范式为 60 秒基线期+120 秒步行任务+120 秒休息期，该范式连续重复 15 次。在数据采集前，嘱咐受试者在跑步机上保持静止站立，并采集 60 秒静止状态下目标脑区的活动情况作为基线数据。在步行任务过程中，连续采集受试者目标脑区的活动情况共计 120 秒。在休息期中，受试者被嘱咐保持静止站立或坐位休息 120 秒，该过程中不采集 fNIRS 数据。针对跨越障碍物行走任务，运动范式为 10 秒基线期+72 秒步行任务，该范式连续重复 9 次。每个步行任务范式包含 4 秒步行任务、10 秒静止期、10 秒休息期。在数据采集前，嘱咐受试者在起点保持静止站立，并采集 10 秒静止状态下目标脑区的活动情况作为基线数据。在跨越障碍物行走和后续静止期中，连续采集受试者目标脑区的活动情况共计 14 秒。在休息期中，受试者被嘱咐回到步行起点并保持静止站立，该过程中不采集 fNIRS 数据。

6. 随访计划：

在治疗结束后的第 12 周（3 个月）和第 24 周（6 个月）随访两次。

7. 统计学分析：

本研究的目标参数包括脊柱侧凸 Cobb 角、临床量表得分、前庭功能指标、椎旁肌结构/功能指标和感觉运动相关脑区的激活水平及其功能连接。在干预前后，采用重复测量方差分析（rmANOVA）对上述目标参数进行统计分析；其中，干预前后时间点为组内因素，设干预条件为组间因素。对于变量间的相关性分析，连续变量采用 Pearson 相关系数检验，有序分类变量或非正态分布变量采用 Spearman 相关系数检验。

8. 不良事件处理：

本研究将无创神经功能评估（fNIRS）、无创神经调控技术（nGVS）和外周本体感觉调控技术（足底震动）应用于 AIS 患者。一般不良事件发生率极低（本人的前期相关研究无不良事件报告）。如果遇到与预期结果相背离的不良事情，研究者会严格尊重伦理要求，按程序及时上报。在收集信息数据过程中也不会对换的人事及心理安全造成风险，为了减少这些风险，我们将按相关规定保密受试者的个人信息，绝不泄露。

9. 数据采集和管理：

本研究计划采用电子化数据采集的方式收集数据。通过数据采集设备附带的 NIRStar 测试台程序、BTS FreeEMG300 数据管理程序、Qualisys Tracker Manager 软件分别录入 fNIRS 数据、EMG 数据、运动学参数数据。所录入到的数据通过 Excel 软件进行汇总和存档。此外，为同步 fNIRS、EMG 和运动学数据，本研究拟使用基于 MATLAB 的代码来整合这些数据。本项目所在实验室的专用电脑对数据进行存储。
