# Supplementary material for: Task-specific effects of noisy galvanic vestibular stimulation on gait symmetry in adolescent idiopathic scoliosis
Source: PeerJ. 2026 Nov 12;14:e21947. doi: 10.7717/peerj.21947 (PMC13770426; doi:10.7717/peerj.21947)
Supplement: Supplementary file 4 [file peerj-14-21947-s001.docx]

**Supplementary Table 1**. Summary of symmetry indices (SI) and normalized cross-correlation coefficients (Xcorr) for kinematic and kinetic parameters.

|  | **GVS conditions** | | |
| --- | --- | --- | --- |
|  | **nGVS** | **Sham** | **No** |
| **Stair descent** |  |  |  |
| SI_ML1 (%) | 27.70±13.56 | 38.35±18.49 | 39.30±18.74 |
| SI_ML2 (%) | 36.89±12.80 | 45.49±16.27 | 47.45±15.91 |
| SI_T (%) | 26.13±10.45 | 32.35±16.57 | 35.86±14.88 |
| SI_α (%) | 16.13±9.87 | 20.28±9.95 | 23.03±9.11 |
| SI_P (%) | 25.33±6.39 | 32.15±10.13 | 33.66±8.72 |
| SI_β (%) | 40.81±15.90 | 47.51±18.55 | 45.31±20.58 |
| SI_θ (%) | 34.61±13.07 | 43.83±12.95 | 45.90±14.01 |
| Xcorr_ML | 0.92±0.05 | 0.83±0.07 | 0.82±0.07 |
| Xcorr_T | 0.88±0.09 | 0.83±0.13 | 0.82±0.12 |
| Xcorr_α | 0.90±0.09 | 0.86±0.09 | 0.84±0.09 |
| Xcorr_P | 0.92±0.06 | 0.87±0.09 | 0.87±0.11 |
| Xcorr_β | 0.89±0.09 | 0.84±0.12 | 0.83±0.11 |
| Xcorr_θ | 0.87±0.08 | 0.83±0.10 | 0.81±0.10 |
| **Level walking** |  |  |  |
| SI_ML1 (%) | 18.74±11.22 | 25.98±16.91 | 28.26±15.67 |
| SI_ML2 (%) | 19.06±9.84 | 21.74±11.99 | 25.64±10.71 |
| SI_T (%) | 11.48±3.28 | 15.95±6.19 | 13.75±5.93 |
| SI_α (%) | 9.24±4.70 | 12.15±6.36 | 13.28±5.55 |
| SI_P (%) | 15.48±6.99 | 21.91±8.70 | 22.50±10.64 |
| SI_β (%) | 24.30±10.42 | 28.77±11.38 | 30.45±11.48 |
| SI_θ (%) | 20.22±7.74 | 25.75±10.82 | 23.97±8.25 |
| Xcorr_ML | 0.97±0.05 | 0.92±0.08 | 0.93±0.05 |
| Xcorr_T | 0.96±0.04 | 0.94±0.04 | 0.93±0.05 |
| Xcorr_α | 0.95±0.03 | 0.93±0.03 | 0.91±0.06 |
| Xcorr_P | 0.96±0.04 | 0.91±0.06 | 0.92±0.06 |
| Xcorr_β | 0.94±0.05 | 0.91±0.07 | 0.90±0.06 |
| Xcorr_θ | 0.94±0.03 | 0.91±0.05 | 0.93±0.04 |
| **Stair ascent** |  |  |  |
| SI_ML1 (%) | 25.35±14.00 | 36.46±20.09 | 39.31±19.91 |
| SI_ML2 (%) | 16.52±11.31 | 21.14±10.76 | 24.03±10.63 |
| SI_T (%) | 21.91±9.62 | 25.91±9.83 | 28.43±12.08 |
| SI_α (%) | 19.73±7.31 | 24.03±9.09 | 26.46±9.23 |
| SI_P (%) | 26.37±10.81 | 31.05±13.25 | 33.17±12.59 |
| SI_β (%) | 33.87±14.05 | 38.87±14.54 | 37.91±14.14 |
| SI_θ (%) | 30.07±12.54 | 38.67±13.06 | 37.03±14.53 |
| Xcorr_ML | 0.96±0.04 | 0.94±0.07 | 0.95±0.07 |
| Xcorr_T | 0.89±0.08 | 0.82±0.13 | 0.85±0.10 |
| Xcorr_α | 0.91±0.06 | 0.86±0.08 | 0.87±0.08 |
| Xcorr_P | 0.93±0.05 | 0.89±0.07 | 0.88±0.10 |
| Xcorr_β | 0.91±0.04 | 0.87±0.07 | 0.86±0.10 |
| Xcorr_θ | 0.93±0.04 | 0.89±0.08 | 0.87±0.09 |

Data were presented as mean±standard deviation. **ML1**: first ground reaction force (GRF) peak in the medial-lateral (ML) direction; **ML2**: second GRF peak in the ML direction; **nGVS**: noisy galvanic vestibular stimulation; **No**: no stimulation; **P**: lateral pelvic displacement; **Sham**: sham stimulation; **T**: lateral trunk displacement; **α**: lateral trunk lean; **β**: pelvic obliquity; **θ**: hip adduction.
