# Supplementary material for: Task-specific effects of noisy galvanic vestibular stimulation on gait symmetry in adolescent idiopathic scoliosis
Source: PeerJ. 2026 Nov 12;14:e21947. doi: 10.7717/peerj.21947 (PMC13770426; doi:10.7717/peerj.21947)
Supplement: Supplementary file 5 [file peerj-14-21947-s002.docx]

**Supplementary Table 2**. Summary of peak values of kinematic and kinetic parameters from convex-side and concave-side limbs across different conditions.

|  | **GVS conditions** | | |
| --- | --- | --- | --- |
|  | **nGVS** | **Sham** | **No** |
| **Stair descent** |  |  |  |
| ML1_convex (%) | 7.34±3.16 | 8.73±3.06 | 8.20±4.41 |
| ML1_concave (%) | 7.11±3.11 | 8.94±3.14 | 8.12±4.15 |
| ML2_convex (%) | 5.88±2.30 | 6.67±2.19 | 6.59±2.77 |
| ML2_concave (%) | 5.59±2.46 | 6.23±2.04 | 6.37±2.68 |
| T_convex (cm) | 12.76±5.46 | 15.95±6.34 | 15.44±6.88 |
| T_concave (cm) | 10.32±4.57 | 11.49±6.07 | 12.62±5.86 |
| α_convex (°) | 8.02±3.59 | 10.24±4.22 | 11.18±4.62 |
| α_concave (°) | 6.69±2.44 | 8.47±3.27 | 9.87±3.33 |
| P_convex (cm) | 14.44±5.72 | 18.82±6.32 | 18.94±5.57 |
| P_concave (cm) | 12.08±3.31 | 15.04±4.69 | 16.40±5.03 |
| β_convex (°) | 11.87±3.41 | 12.22±3.47 | 14.49±3.34 |
| β_concave (°) | 14.17±6.20 | 16.67±5.52 | 18.59±5.81 |
| θ_convex (°) | 12.60±4.96 | 16.05±5.63 | 15.73±4.73 |
| θ_concave (°) | 17.53±6.42 | 19.35±6.47 | 19.13±5.15 |
| **Level walking** |  |  |  |
| ML1_convex (%) | 6.31±2.95 | 7.58±3.42 | 7.82±2.51 |
| ML1_concave (%) | 5.22±3.01 | 6.61±3.64 | 8.11±2.94 |
| ML2_convex (%) | 4.93±2.10 | 5.83±2.76 | 6.01±2.42 |
| ML2_concave (%) | 5.47±3.71 | 6.57±3.37 | 6.15±2.19 |
| T_convex (cm) | 8.24±3.87 | 12.07±5.22 | 11.46±5.45 |
| T_concave (cm) | 6.33±3.01 | 9.75±3.78 | 10.19±4.33 |
| α_convex (°) | 5.98±2.09 | 7.51±3.31 | 8.05±3.25 |
| α_concave (°) | 4.32±1.63 | 6.28±2.64 | 7.33±2.93 |
| P_convex (cm) | 10.25±5.39 | 13.02±4.96 | 13.18±3.83 |
| P_concave (cm) | 9.91±3.04 | 11.02±3.19 | 11.17±3.90 |
| β_convex (°) | 10.51±3.41 | 11.75±3.86 | 11.33±4.03 |
| β_concave (°) | 15.83±5.55 | 16.58±5.54 | 16.77±5.93 |
| θ_convex (°) | 11.74±4.13 | 15.58±5.00 | 15.71±5.22 |
| θ_concave (°) | 16.12±5.54 | 18.60±5.54 | 18.90±5.14 |
| **Stair ascent** |  |  |  |
| ML1_convex (%) | 8.32±3.66 | 9.61±4.61 | 9.77±3.49 |
| ML1_concave (%) | 8.28±3.48 | 9.35±4.01 | 9.72±3.47 |
| ML2_convex (%) | 6.32±2.39 | 7.89±3.25 | 8.53±2.17 |
| ML2_concave (%) | 6.68±2.32 | 7.66±3.07 | 8.44±2.15 |
| T_convex (cm) | 12.08±6.03 | 14.11±6.47 | 12.44±6.14 |
| T_concave (cm) | 9.82±4.81 | 13.02±6.24 | 16.00±7.49 |
| α_convex (°) | 10.10±4.48 | 11.45±4.89 | 11.76±4.49 |
| α_concave (°) | 7.76±2.92 | 8.51±3.55 | 9.51±4.10 |
| P_convex (cm) | 13.02±5.62 | 17.99±4.86 | 18.05±6.68 |
| P_concave (cm) | 11.03±3.58 | 15.71±6.86 | 16.33±3.88 |
| β_convex (°) | 11.73±3.92 | 12.25±3.97 | 13.56±3.66 |
| β_concave (°) | 14.84±6.02 | 15.77±5.22 | 17.32±6.46 |
| θ_convex (°) | 12.62±5.05 | 15.14±4.55 | 16.02±4.15 |
| θ_concave (°) | 18.47±5.25 | 19.67±6.49 | 20.20±4.96 |

Data were presented as mean±standard deviation. **ML1**: first ground reaction force (GRF) peak in the medial-lateral (ML) direction; **ML2**: second GRF peak in the ML direction; **nGVS**: noisy galvanic vestibular stimulation; **No**: no stimulation; **P**: lateral pelvic displacement; **Sham**: sham stimulation; **T**: lateral trunk displacement; **α**: lateral trunk lean; **β**: pelvic obliquity; **θ**: hip adduction.
