# Supplementary material for: Task-specific effects of noisy galvanic vestibular stimulation on gait symmetry in adolescent idiopathic scoliosis
Source: PeerJ. 2026 Nov 12;14:e21947. doi: 10.7717/peerj.21947 (PMC13770426; doi:10.7717/peerj.21947)
Supplement: Supplementary file 6 [file peerj-14-21947-s003.docx]

**Supplementary Table 3**. Summary of symmetry indices (SI) for kinematic parameters without static offset correction and respective statistical results from the two-way repeated-measures ANOVA for sensitivity analysis.

|  | **Tasks** | **Stimulation conditions** | | | **Effect** | ***F*** | ***df_effect_, df_error_*** | ***P*** | ***η^2^_p_*** |
| --- | --- | --- | --- | --- | --- | --- | --- | --- | --- |
|  |  | **nGVS** | **Sham** | **No** |  |  |  |  |  |
| SI_T (%) | Stair descent | 32.53±17.55 | 40.11±22.29 | 46.62±25.84 | Interaction | 0.828 | 3.12, 90.48 | 0.482 | 0.028 |
|  | Level walking | 16.64±9.52 | 20.67±12.73 | 22.78±14.98 | Stimulation | 1.371 | 1.62, 46.98 | 0.248 | 0.045 |
|  | Stair ascent | 31.55±16.45 | 39.73±20.72 | 38.15±22.49 | Task | 1.879 | 1.48, 42.92 | 0.178 | 0.061 |
| SI_α (%) | Stair descent | 23.74±12.99 | 35.88±19.97 | 34.52±18.70 | Interaction | 1.340 | 4, 116 | 0.257 | 0.044 |
|  | Level walking | 17.77±11.24 | 27.62±17.21 | 25.15±16.52 | Stimulation | 5.309 | 2, 58 | 0.008** | 0.155 |
|  | Stair ascent | 25.06±15.98 | 32.99±20.52 | 35.08±19.33 | Task | 2.928 | 2, 58 | 0.061 | 0.092 |
| SI_P (%) | Stair descent | 39.54±21.21 | 41.15±25.35 | 44.05±23.59 | Interaction | 2.283 | 3.44, 99.76 | 0.063 | 0.073 |
|  | Level walking | 27.73±14.76 | 35.46±21.15 | 35.21±23.61 | Stimulation | 2.420 | 1.76, 51.04 | 0.098 | 0.077 |
|  | Stair ascent | 35.78±20.24 | 37.66±23.55 | 42.82±27.30 | Task | 1.904 | 1.64, 47.56 | 0.158 | 0.062 |
| SI_β (%) | Stair descent | 49.18±27.94 | 55.63±30.88 | 67.51±38.85 | Interaction | 2.308 | 2.96, 85.84 | 0.106 | 0.074 |
|  | Level walking | 32.80±19.45 | 42.12±25.19 | 45.27±23.38 | Stimulation | 3.803 | 1.58, 42.82 | 0.058 | 0.123 |
|  | Stair ascent | 43.71±23.71 | 60.32±32.32 | 66.01±36.01 | Task | 2.158 | 1.60, 46.40 | 0.149 | 0.069 |
| SI_θ (%) | Stair descent | 31.65±17.92 | 38.13±17.23 | 44.80±21.23 | Interaction | 2.025 | 2.76, 80.04 | 0.138 | 0.065 |
|  | Level walking | 26.78±16.11 | 28.11±19.75 | 32.08±19.30 | Stimulation | 0.723 | 1.46, 42.34 | 0.400 | 0.024 |
|  | Stair ascent | 35.69±21.58 | 40.99±23.63 | 43.70±27.49 | Task | 1.566 | 1.50, 43.50 | 0.217 | 0.051 |

Data were presented as mean±standard deviation. ***df***: degrees of freedom; **nGVS**: noisy galvanic vestibular stimulation; **No**: no stimulation; **P**: lateral pelvic displacement; **Sham**: sham stimulation; **T**: lateral trunk displacement; **α**: lateral trunk lean; **β**: pelvic obliquity; **θ**: hip adduction. **: *p*<0.01.
